# Associations between constructs related to social relationships and mental health conditions and symptoms: an umbrella review

**DOI:** 10.1186/s12888-023-05069-0

**Published:** 2023-09-04

**Authors:** Eiluned Pearce, Mary Birken, Sarah Pais, Millie Tamworth, Yutung Ng, Jingyi Wang, Beverley Chipp, Ellena Crane, Merle Schlief, Jinyan Yang, Aggelos Stamos, Lui Kwan Cheng, Maria Condon, Brynmor Lloyd-Evans, James B. Kirkbride, David Osborn, Alexandra Pitman, Sonia Johnson

**Affiliations:** 1 Division of Psychiatry, University College London, London, UK; 2 Key Laboratory of Public Health Safety, NHC Key Laboratory of Health Technology Assessment, School of Public Health, Fudan University, Shanghai, China; 3 Co-production Group, Loneliness and Social Isolation in Mental Health Research Network, Division of Psychiatry, University College London, London, UK; 4 Camden and Islington NHS Foundation Trust, London, UK

**Keywords:** Loneliness, Social isolation, Social network, Mental Health, Umbrella review

## Abstract

**Background:**

Loneliness and social isolation are increasingly recognised as prevalent among people with mental health problems, and as potential targets for interventions to improve quality of life and outcomes, as well as for preventive strategies. Understanding the relationship between quality and quantity of social relationships and a range of mental health conditions is a helpful step towards development of such interventions.

**Purpose:**

Our aim was to give an overview of associations between constructs related to social relationships (including loneliness and social isolation) and diagnosed mental conditions and mental health symptoms, as reported in systematic reviews of observational studies.

**Methods:**

For this umbrella review (systematic review of systematic reviews) we searched five databases (PsycINFO, MEDLINE, EMBASE, CINAHL, Web of Science) and relevant online resources (PROSPERO, Campbell Collaboration, Joanna Briggs Institute Evidence Synthesis Journal). We included systematic reviews of studies of associations between constructs related to social relationships and mental health diagnoses or psychiatric symptom severity, in clinical or general population samples. We also included reviews of general population studies investigating the relationship between loneliness and risk of onset of mental health problems.

**Results:**

We identified 53 relevant systematic reviews, including them in a narrative synthesis. We found evidence regarding associations between (i) loneliness, social isolation, social support, social network size and composition, and individual-level social capital and (ii) diagnoses of mental health conditions and severity of various mental health symptoms. Depression (including post-natal) and psychosis were most often reported on, with few systematic reviews on eating disorders or post-traumatic stress disorder (PTSD), and only four related to anxiety. Social support was the most commonly included social construct. Our findings were limited by low quality of reviews and their inclusion of mainly cross-sectional evidence.

**Conclusion:**

Good quality evidence is needed on a wider range of social constructs, on conditions other than depression, and on longitudinal relationships between social constructs and mental health symptoms and conditions.

**Supplementary Information:**

The online version contains supplementary material available at 10.1186/s12888-023-05069-0.

## Introduction

Evidence is accumulating on the effects of social relationships, or of the lack of them, on physical and mental health. Loneliness and social isolation have been associated with increased mortality rates in two meta-analytic reviews, with comparable effect sizes to those observed for smoking, obesity, and physical inactivity as risk behaviors [1, 2]. Loneliness and social isolation are longitudinally associated with the development of cardiovascular disease [3], elevated blood pressure [4, 5], and increased fatigue and pain [6]. Among people with mental health problems, loneliness and social isolation are more prevalent than in the general population [7, 8]. Associations with loneliness and social isolation have been reported for depressive disorders [9, 10] and symptoms [11], self-harm [12], psychosis [13, 14], being diagnosed with a “personality disorder” [15], cognitive decline [16, 17], mild cognitive impairment, and dementia [18]. In a rapidly expanding research field, an up-to-date synthesis is needed of evidence on whether, and in what ways, loneliness, social isolation, and related constructs are associated with the incidence and prevalence of a range of mental health conditions, and with their outcomes.

Several other social constructs are related to loneliness and social isolation, including social support, social networks, individual social capital, confiding relationships, connectedness and alienation. Wang et al. [19] have proposed a conceptual model to incorporate these constructs related to social relationships at the individual level in mental health research. According to this model, these constructs can be grouped as: (i) perceived or subjective experiences of social relationships (such as loneliness, perceived social isolation, social support, confiding relationships or individual-level social capital); (ii) objective aspects of social isolation (such as the number of social contacts and social network size), or (iii) constructs that combine measures of both quality and quantity of social relationships (such as social support from members of an individual’s social network).

Several systematic reviews have been published regarding associations between constructs related to social relationships and aspects of mental health, e.g. [10, 20–23]. However, these reviews have typically focused on specific mental health outcomes in particular populations, so that they do not provide a holistic stock-take of the overall state of evidence in this field, its implications for research, policy and practice, and the gaps still to be addressed.

Umbrella reviews, which are systematic reviews of the systematic review evidence [24], can inform policy, practice and further research by providing a systematic overview of current evidence and its gaps. One recent umbrella review explored the associations between loneliness and outcome measures related to mental health [25]. This concluded that loneliness has a range of adverse impacts on mental and physical health outcomes, but it did not include other related constructs such as social isolation, or reviews of associations between loneliness and social isolation and mental health diagnoses. To our knowledge, there is no umbrella review that synthesises the evidence for associations between a comprehensive set of constructs related to social relationships and specific mental health problems. Such a review has potential value in allowing policy makers, clinicians and researchers to identify areas in which there is a robust and actionable body of evidence regarding connections between mental health conditions and social relationships, and those in which there is a pressing need for more evidence.

This umbrella review addresses this gap by providing an updated and comprehensive overview of the evidence on the associations between a full range of social constructs at the individual level (Table 1) and mental health diagnoses and symptoms in both clinical and population-based samples. The constructs we used to encapsulate important aspects of social relationships including loneliness and isolation are those identified by Wang et al. [19] in their conceptual review. We aimed to address the following linked research questions:

1. What is the evidence from systematic reviews regarding associations in general population samples between constructs related to social relationships, and presence of mental health conditions and symptoms?
2. What evidence is there from systematic reviews regarding associations between constructs related to social relationships and severity of psychiatric symptoms among people diagnosed with mental health conditions?
3. What evidence is there from systematic reviews of longitudinal relationships between constructs related to social relationships and risk of onset of mental health conditions in the general population?

**Table 1:** Definitions of constructs related to social relations

Construct	Description	Reference (all reviewed in Wang et al., 2017 [19])
Loneliness	A painful subjective emotional state occurring when there is a discrepancy between desired and achieved patterns of social interaction.	Hawkley & Cacioppo, 2009; Peplau & Perlman, 1982
Social isolation	Inadequate quality and quantity of social relations with other people at the individual, group, community, and larger social environment levels where human interaction takes place.	Zavaleta, Samuel & Mills, 2014
Social support	*Structural* social support: the existence, quantity, and properties of an individual’s social relations; it focuses on the study of structural aspects of social contacts, such as the characteristics of the support network. *Functional* social support: the functions fulfilled by social relations, such as emotional support which involves caring, love and empathy; instrumental support (referred to by many as tangible support); informational support which consists of information, guidance or feedback that can provide a solution to a problem; appraisal support which involves information relevant to self-evaluation and social companionship, which involves spending time with others in leisure and recreational activities.	Cohen & Hoberman, 1983; House 1981; Moreno, 2004; Wills, 1985
Social network	A specific set of linkages among a defined set of persons, with the additional property that the characteristics of these linkages as a whole may be used to interpret the social behaviour of the persons involved: *Size*: the number of people with whom the respondent has had social contact e.g. in the last month; *Frequency of contact*: the number of people with whom the respondent has had social contact e.g. daily; weekly; or monthly over the past month; *Density*: the proportion of all possible ties between network members which are present (i.e., how many of a respondent’s network know each other); *Proportion of kin/non-kin* in social network: How many of the total number of people within a respondent’s social network are relatives?; *Intensity*: whether relationships are “uniplex” (one function only) or “multiplex” (more than one function); *Directionality*: who is helping whom in a dyadic relationship.	Cohen & Sokolovsky, 1978; Mitchell, 1969
Individual social capital	A series of resources that individuals earn as a result of their membership in social networks, and the features of those networks that facilitate coordination and cooperation for mutual benefit; can be understood as the property of an individual. Most commonly measured by asking individuals about their participation in social relationships (such as membership of groups) and their perceptions of the quality of those relationships.	De Silva, McKenzie, Harpham & Huttly, 2005; McKenzie, Whitley & Weich, 2002; Portes, 1998; Putnam, 2000
Confiding relationships	The number of people with whom the respondent reports they can talk about worries or feelings.	Brown & Harris, 1978; Murphy, 1982

## Methods

**Fig. 1:**
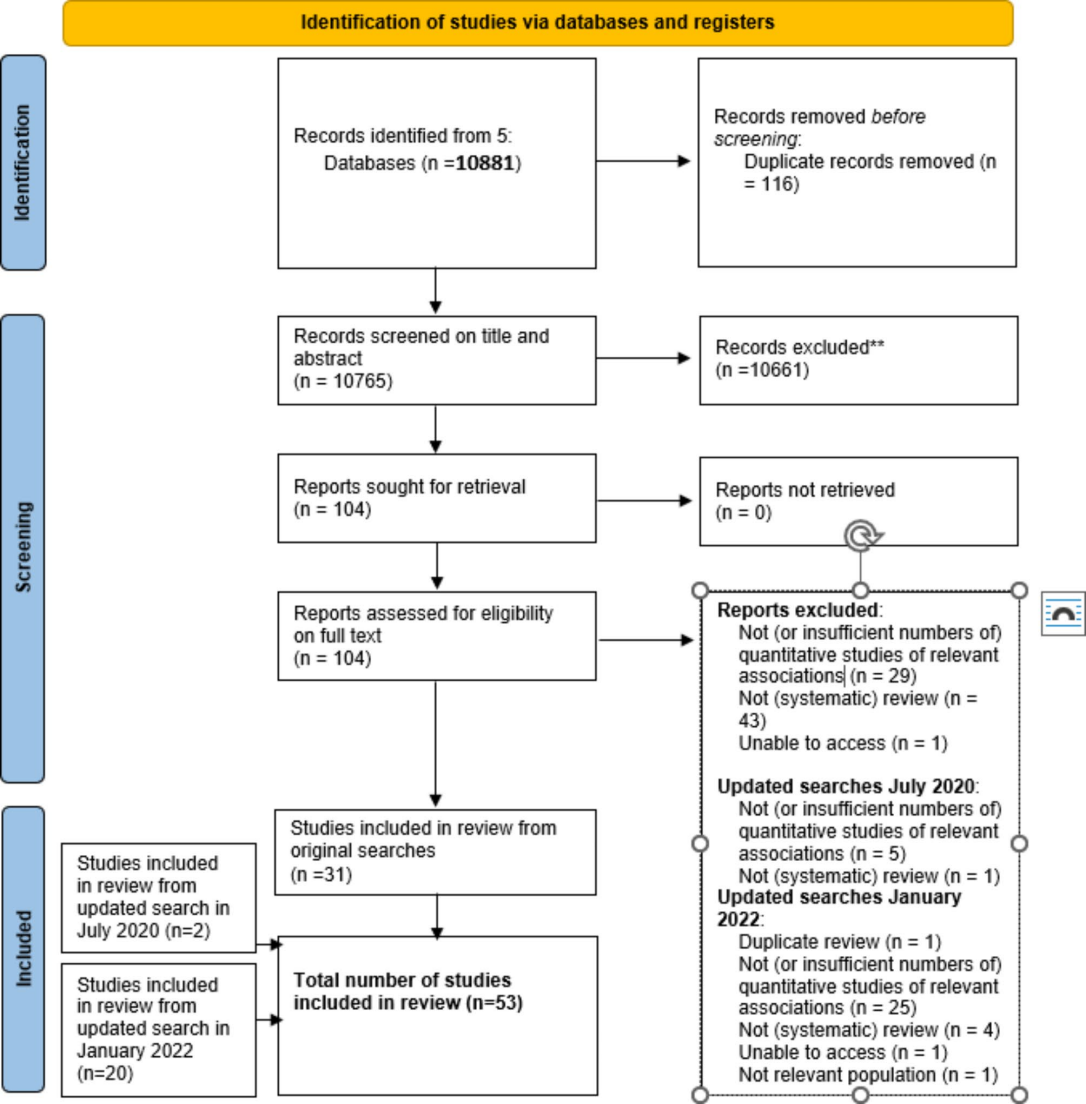
Prisma diagram identification of studies via databases and registers *From:* Page MJ, McKenzie JE, Boosuvt PM, Boutron I, Hoffmann TC, Mulrow CD, et al. The PRISMA 2020 statement an updated guideline for reporting systematic reviews. BMJ 2021;372:n71. Doi:1136/bmj.n71For more information, visit: http://www.prisma-statement.org/

We followed Preferred Reporting Items for Systematic Reviews and Meta-Analyses (PRISMA) guidelines (Fig. 1). The protocol for this review was pre-registered with the international Prospective Register of Systematic Reviews.

(PROSPERO: https://www.crd.york.ac.uk/prospero/display_record.php?ID=CRD42020192509).

### Inclusion and exclusion criteria

#### Included exposures: measures related to social relationships

We included the subjective aspects of social relationships and objective measures of social network size and structure identified in the conceptual review by Wang et al. [19] to encapsulate the main dimensions of social relationships assessed in mental health research. Table 1 summarises and defines the included domains of measurement. We excluded dimensions of social relationships beyond the individual level, such as ecological social capital or social exclusion, following Wang et al. [19].

#### Included outcomes: mental health measures

We considered a full range of mental health diagnoses and psychiatric symptoms, but we excluded neurodegenerative diagnoses (e.g., dementia), neurodevelopmental disorders (e.g., intellectual disabilities), general wellbeing outcomes and suicide-related outcomes, as well as cohorts of people selected on the basis of a primary physical health diagnosis.

#### Included methods

We included reviews of quantitative studies (cross-sectional and longitudinal) of associations between constructs related to social relationships (exposures; see above) and mental health diagnoses or psychiatric symptom severity (outcomes) in clinical and general population samples. We included meta-analyses, narrative systematic reviews and any other literature reviews that followed systematic methods. Included reviews varied in whether they reported adjusted and/or unadjusted associations. We excluded individual empirical studies and reviews that were not systematic. We did not apply any restrictions by publication date, language or age to our search.

### Search strategy

We searched PsycINFO, MEDLINE, EMBASE, CINAHL, and Web of Science databases. We also searched online repositories of systematic reviews: PROSPERO, Campbell Collaboration, and the Joanna Briggs Institute Evidence Synthesis Journal.

The following search terms related to social isolation and loneliness were used following the conceptual review by Wang et al. [19]: social isolation OR loneliness OR social network* OR social support OR confiding OR confide OR social contact* OR social relation* OR social capital.

The above terms were combined with the following search terms for mental health problems and symptoms:

(mental OR psychiatr* OR schizo* OR psychosis OR psychotic OR depress* OR mania* OR manic OR bipolar near/5 (disorder or disease or illness) OR anxiety) OR (Eating Disorder* OR Anorexia Nervosa OR Bulimia Nervosa OR Binge Eating Disorder) OR personality disorder* OR borderline personality OR emotionally unstable personality OR histrionic personality or narcissistic personality OR antisocial personality OR paranoid personality OR schizoid personality OR schizotypal personality OR avoidant personality OR dependent personality OR obsessive compulsive personality).

The search strategy for Medline appears in full in Supplementary Table S1; this was adapted to other search engines. The results of all searches were imported into EndNote. The initial search was run in August 2019 with updates in July 2020 and January 2022. Following removal of duplicate citations, a reviewer (MB or YN) screened the abstracts and titles of all articles against the inclusion criteria, and second reviewers (EP, MT) randomly screened 10% of included abstracts to check agreement. MB, EP, YN and MT screened the full texts of selected articles, with all full texts screened by a second reviewer. AP and EP discussed any disagreements and clarified criteria to achieve good consistency. Data were extracted from included reviews independently by nine reviewers (MB, EP, JY, EC, AS, LKC and MC, YN and MT): senior researchers MB and EP supervised the other reviewers to ensure consistency and checked extracted data.

For included studies, we developed and piloted a pro forma that allowed extraction of data on setting(s), objectives of the review, type of review, inclusion criteria, number of included studies, publication date range for included studies, type of analysis, and outcomes reported relating to mental health and to loneliness, social isolation and related constructs.

### Quality assessment

Seven reviewers (JY, EC, AS, LKC, MC, MT and YN) independently assessed the methodological quality of included studies under the supervision of MB and EP, using a modified version of AMSTAR (A Measurement Tool to Assess Systematic Reviews) (Supplementary Table S2). One reviewer (MT) independently quality rated all included reviews to ensure consistency, and two further reviewers (YD, MH) then checked these again. The AMSTAR-2 tool has 16 items in total and enables appraisal of systematic reviews. Item 7, which assesses adequacy of explanations for excluding studies from reviews at the full text stage was modified (reviews were rated as a “no” but not as critically flawed if they included some explanations for exclusion but not a full list, and as critically flawed if no explanation was given) and Items 3, 10 and 16 (on interventions, funding and conflict of interest) were excluded because of limited relevance or feasibility. We included rapid systematic reviews and scoping reviews if the searches and data extraction were systematic.

### Data synthesis

We conducted a narrative synthesis: we grouped reviews according to disorder or symptom type, and within these categories we separated them by different social constructs. Social constructs were grouped following Wang et al’s typology. Reviews may appear in more than one section of the narrative synthesis if they included multiple social constructs e.g. both loneliness and social support. Most reviews focused on broad groups of mental health conditions, such as depressive illnesses, conditions related to anxiety, perinatal conditions and psychosis: we were able to categorise the reviews straightforwardly by grouping together those reporting that they focused on the same, or similar mental health conditions. In our narrative, we distinguish reviews that report results from meta-analysis, and those that are longitudinal rather than reporting on cross-sectional associations. Where possible, we distinguished evidence on symptom severity versus diagnosis.

## Results

**Table 2:** Characteristics of included studies

Author & year	Type of review	Geographical setting of included studies	No. of relevant primary studies	Population	Year of included studies	Type of analysis	Exposure	Outcome	Quality rating/ overall confidence
**Depression – loneliness**									
Cohen-Mansfield et al., (2016)	Systematic Review	Finland, USA, Netherlands, Spain, Ireland, UK	12	Older Adults	2004–2012	Narrative Synthesis	Loneliness	‘Depression’ including studies measuring depressive symptoms, low mood, ‘uselessness’, ‘lack of taking initiative’, and ‘expecting negative reactions from caregivers’	Critically low
Erzen & Çikrikci (2018)	Systematic Review	Not reported	70^a^	Adults	1980–2018	Meta-analysis	Loneliness	Depression (general term “depression”)	Critically low
**Depression – loneliness, social isolation, social support and social network size**									
Choi et al., (2015)	Systematic Review	Malaysia, USA, New Zealand, Turkey, Mexico	8	Older Adults	2003–2014	Narrative Synthesis	Subjective and objective social isolation	Depression (symptoms)	Critically low
Courtin & Knapp et al., (2017)	Scoping Review	15 countries overall, including western Europe (UK and Netherlands) and USA	29	Older Adults	2000–2013	Narrative Synthesis	Loneliness and social isolation	Depression	Critically low
Worrall et al., (2020)	Systematic Review	Not reported	37	Older Adults	2007–2018	Narrative Synthesis	Loneliness, Social Support & Social Network	Depressive symptom (severity)	Low
**Depression – social support and/or social networks**									
Edwards et al., (2020)	Systematic Review	Germany, Columbia, USA	13	Active Christian clergy-members (7.6% United Methodist; 39.2% Catholic; 12.2% Presbyterian; 1% other Protestant denominations)	1997–2019	Narrative Synthesis	Social Support	Depression (no specific clarification about whether depression diagnosis or symptoms)	Critically low
Gariépy et al., (2016)	Systematic Review	USA, Canada and Europe, Australia and New Zealand	100	All ages in general population	1988–2014	Meta-analysis	Social Support	Depression (depression diagnosis or depressive symptoms)	Critically low
Guo & Stensland et al., (2018)	Systematic Review	USA	32	Older Chinese and Korean immigrants in USA	1996–2016	Narrative Synthesis	Social Support & Social Networks	Depression (depressive symptoms)	Critically low
Guruge et al., (2015)	Scoping Review	Canada	34	Immigrant women youth	1990–2013	Narrative Synthesis	Social Support	Depression (depressive symptoms) (n = 18 studies), or determinants of mental health in general (n = 16)	Critically low
Hall et al., (2018)	Systematic Review	United States	7	LGBQ adolescents (15–24 years of age)	2000–2013	Narrative Synthesis	Social Support (from friends and family, sexuality specific social support)	Depression (symptom severity or threshold for clinically significant depressive symptoms)	Critically low
Mohd et al., (2019)	Systematic Review	China, Japan, Singapore, Taiwan, Hong Kong, Korea, Macau, Thailand	24	Older adults living in Asia	2001–2016	Narrative Synthesis	Structural and functional social support (includes social network size as an aspect of structural social support)	Depression (self-reported or diagnosed)	Critically low
Qiu et al., (2020)	Systematic Review	China	4	Chinese adults > age 55	2005–2019	Meta-analysis	Social Support	Clinician diagnosis of depression	Critically Low
Rueger et al., (2016)	Meta-analytic Review	Finland, Israel, Romania, Turkey, Belgium, Brazil, Burundi, Hong Kong, Iceland, Israel, Italy, Kenya, Korea, Mexico, Russia (k = 2 each); Austria, Croatia, France, Germany, Greece, Hungary, Japan, Kuwait, Poland, Portugal, Puerto Rico, South Africa, Spain, Sweden, Switzerland, United Arab Emirates, United Kingdom, Australia, United States	341	Children and adolescents	1983–2014	Meta-analysis	Social Support	Depression (diagnosis, symptoms)	Low
Santini et al., (2015)	Systematic Review	USA, Asia, Europe	50	Adults from general population	2004–2013	Narrative Synthesis	Social Support & Social Networks	Depression (symptoms, presence, onset or development)	Low
Schwarzbach et al., (2014)	Systematic Review	USA, Asia, Europe	17	Older adults	1985–2012	Narrative Synthesis	Social Support & Social Networks	Depression (dimensional diagnosis, prevalence, incidence)	Critically low
Visentini et al., (2018)	Systematic Review	Finland, Hungary, India, Netherlands, Norway, Sweden, USA	18	Patients with chronic depression	1986–2015	Narrative Synthesis	Social Support & Social Networks	Chronic Depression (diagnosis)	Low
**Perinatal/postnatal depression & anxiety - social support**									
Bayrampour et al., (2018)	Systematic Review	USA, Canada, Hungary, Turkey, Hong Kong, New Zealand, Poland, Germany, Bangladesh, UK, South Africa, Greece	22	Pregnant women	1982–2015	Meta-analysis, Narrative Review	Social Support	Anxiety (symptoms, generalised anxiety, overall anxiety disorders in pregnancy)	Low
Bedaso et al., (2021)	Systematic Review	Jordan, Nigeria, Italy, UK, Indonesia, Canada, Ethiopia, Jamaica, Turkey, Singapore, USA, China, Hungary, Greece, Germany, South Africa, Hong Kong, India, Finland, Malaysia, Sweden, Iran, Malawi, Pakistan, Bangladesh, Taiwan, Tanzania	63	Adult pregnant mothers	2004–2019	Meta-analysis, Narrative Review	Social Support	Any diagnosed depressive disorders and general anxiety disorder according to ICD and DSM or depressive disorders and general anxiety disorder based on a valid screening tool	Moderate
Desta et al., (2021)	Systematic Review	SNNPR, Oromia, Amhara, Harar, Addiss (regions within Ethiopia)	4	Postpartum women with postpartum depression in Ethiopia	2018–2020	Meta-analysis	Social Support	Postpartum depression	Critically low
Nisar et al., (2020)	Systematic Review	23 regions of Mainland China	10	Chinese women in perinatal period	1996–2018	Meta-analysis	Social Support	Perinatal depression	Moderate
Qi et al., (2021)	Systematic Review	China	9	Chinese women who have given birth to at least one child including those living in countries other than China	2004–2020	Meta-analysis	Social Support	Onset of post-partum depression	Critically Low
Razurel et al., (2013)	Systematic Review	UK, Canada, Taiwan, Israel, Australia and USA	25	Perinatal mothers	2000–2009	Narrative Synthesis	Social Support	Postnatal depression (symptoms)	Critically low
Tarsuslu et al., (2020)	Systematic Review	Global	5	Fathers post-partum	2010–2019	Narrative Synthesis	Social Support	Post-partum depression measured on differing scales but established ones.	Critically Low
Tolossa et al., (2020)	Systematic Review	Ethiopia	5	Women in postpartum period with all studies conducted in Ethiopia	2016–2019	Meta-analysis, Narrative Review	Social Support	Secondary outcome was to determine the main risk factors associated with PND with social support being one of the risk factors examined.	Low
Zeleke et al., (2021)	Systematic Review	Ethiopia	3	Postnatal mothers	2010–2020	Meta-analysis, Narrative Review	Social Support	Diagnosis of PND. Does not specify if related to either onset or severity, just ‘more likely to have it’	Low
**Depression, anxiety, and OCD – loneliness and social isolation or social support or social capital**									
Gilmour et al., (2020)	Systematic Review	United States, South Korea, Portugal, Hong Kong, Belgium, Taiwan	5	Young adults	2011–2018	Narrative Synthesis	Social Support	Mental health conditions; Depression and anxiety	Low
Loades et al., (2020)	Rapid Systematic Review	USA, China, Europe and Australia, India, Malaysia, Korea, Thailand, Israel, Iran, and Russia	63	Children/adolescents	1983–2020	Narrative Synthesis	Isolation	Depression & anxiety (symptom severity)	Moderate
Mahon et al., (2006)	Meta-analytic Review	Not reported	30	Adolescents (11–23 years of age)	1980–2004	Meta-analysis	Loneliness	Depression (general term “depression”) and ‘social anxiety’, Social support	Critically low
**Anxiety, and ADHD - loneliness**									
Hards et al., (2021)	Rapid Systematic Review	United States, Australia, Canada, China, Taiwan	8	Predominantly children or adolescents with heightened distress, mental health problems, or diagnoses based on internationally recognized (DSM or ICD)	1993–2020	Narrative Synthesis	Loneliness	Depression, anxiety, and obsessive–compulsive disorder (OCD)	Low
**Anxiety – social support and/or social network**									
Zimmermann et al., (2020)	Systematic Review	Global	4	Adults in general population who had a clinically diagnosed anxiety disorder. They looked at anxiety disorders across the spectrum (outcomes included OCD, Specific Phobia, SAD, GAD etc.)	2010–2014	Narrative Synthesis	Measure of social support not defined at top level; underlying studies either measured social support (via scales) with one study looking at quantity of social network and experience of close friendships	Social support was looked at in relation to social anxiety disorder (2 studies) and as a risk factor for any anxiety disorder (1 study)	Moderate
**PTSD – social support**									
Allen et al., (2021)	Systematic Review	Global but predominately USA and China	50	Child and adolescents	1996–2019	Meta-analysis	Social Support	Primary aim was to evaluate the overall relationship between social support and PTSD; secondary aim was to investigate the relationship between severity of PTSD relative to levels of social support	Moderate
Blais et al., (2021)	Meta-analytic Review	U.S.	38	Service members or veterans in the U.S. military	1998–2019	Meta-analysis	Types of Social support (perceived, enacted, structural, or social negativity); Sources of social support (military v. non-military); timing of social support (during deployment v. not during deployment)	Severity of PTSD symptoms	Moderate
Tirone et al., (2021)	Systematic Review	Primarily USA	29	Adult betrayal trauma survivors	2000–2019	Meta-analysis	Social Support	PTSD symptom severity (understanding the moderators of)	Critically Low
Trickey et al., (2012)	Meta-analytic Review	UK, USA	4	Children and adolescents (6–18 years)	1996–2007	Meta-analysis	Low social support (including feeling isolated/ excluded and perceived alienation)	PTSD symptom severity and diagnosis	Critically Low
Zalta et al., (2021)	Systematic Review	Global	150	All participants had to be exposed to a DSM-5 criterion event and had to be > 18 years; treatment studies were excluded and study population chosen on basis of their PTSD were excluded	1980–2019	Meta-analysis	Social support defined as negative social reactions; perceived level of support’ structural support; enacted support	PTSD symptom severity	Moderate
**PTSD and Depression – social support and/or social network**									
Scott et al., (2020)	Systematic Review	Global	10	Adults 18 years plus following bereavement after sudden / violent death	1988–2019	Narrative Synthesis	Informal Social Support	Relevant outcome to this was a psychiatric symptoms (including depression and PTSD) either clinical diagnosis or measure of symptom severity	Moderate
**Psychosis – loneliness**									
Chau et al., (2019)	Meta-analytic Review	USA, UK, Netherlands, Poland, Israel, USA, Germany, Ireland, Denmark, France, Australia	31	Adults with and without clinical diagnosis of psychosis	1995–2018	Meta-analysis	Loneliness	Psychosis (positive and negative symptoms, psychotic experiences)	Low
Michalska da Rocha et al., (2018)	Systematic Review	United States, Great Britain, Australia, Germany, Israel, Poland	13	People with a diagnosis of a psychotic disorder	1993–2016	Meta-analysis	Loneliness	Psychosis (symptoms)	Moderate
Lim et al., (2018)	Systematic Review	Ireland, Israel, Philippines, Poland, UK, USA, Serbia	9	People with a diagnosis of a psychotic disorder	1995–2016	Narrative Synthesis	Loneliness	Psychosis (diagnosis)	Moderate
**Psychosis – social support and/or social networks**									
Degnan et al., (2018)	Systematic Review	USA, UK, Poland, Australia, Denmark, Austria	16	Adults with schizophrenia	1989–2013	Meta-analysis, Narrative Review	Social Networks	Schizophrenia (symptomatic and/or functional outcome)	Moderate
Gayer-Anderson and Morgan (2013)	Systematic Review	North America, Europe, Australia, Korea	38	People aged 16–64 years, first episode psychosis and general population samples with psychotic experiences or schizotypal traits	1976–2011	Narrative Synthesis	Social Support & Social Networks	Early psychosis (first episode)	Critically low
Palumbo et al., (2015)	Systematic Review	USA, Austria, Belgium, Denmark, Finland, Germany, Poland, Spain, UK, Nigeria, Brazil	20	Adults with a diagnosed psychotic disorder	1976–2013	Narrative Synthesis	Social Networks	Psychosis (a standardised diagnosis of either schizophrenia, schizoaffective disorder, “narrow schizophrenia” spectrum disorder, or “psychosis”)	Critically low
**Bipolar disorder – social support**									
Greenberg et al., (2014)	Systematic Review	Not reported	15	Adults with bipolar disorder	1985–2010	Narrative Synthesis	Social Support	Bipolar disorder (general term “bipolar disorder”, manic or depressive episodes)	Critically low
Studart et al., (2015)	Systematic Review	Not reported	13	People with bipolar disorder	1985–2012	Narrative Synthesis	Social Support	Bipolar disorder (general term “bipolar disorder”, symptoms, mania and depression)	Critically low
**Depression, Bipolar, Psychosis & Anxiety disorders – loneliness & perceived social support**									
Wang et al., (2018)	Systematic Review	North America, Europe, Israel	34	Adults with mental illnesses	1988–2016	Narrative Synthesis	Social Isolation, Social Support	Depression, bipolar, schizophrenia/schizoaffective, anxiety, mixed mental illness (relapse, measures of functioning or recovery, symptom severity, global outcome)	Moderate
**Eating disorders – social support**									
Arcelus et al., (2013)	Systematic Review	Not reported	4	Patients with eating disorders (anorexia and/or bulimia), along with non-clinical populations (university students) and controls	1992–1999	Narrative Synthesis	Social Support	Eating disorders (symptom severity)	Low
**Mental ill-health in general (no specified mental health condition) – social support**									
Casale & Wild, (2013)	Systematic Review	Not reported	17	Adult caregivers who have HIV and adult caregivers caring for HIV/AIDS affected children	1995–2010	Narrative Synthesis	Social Support	Mental Health outcomes	Critically low
Tajvar et al., (2013)	Systematic Review	Middle Eastern Countries	9	Older Adults	1986–2010	Narrative Synthesis	Social Support	Mental Health outcomes	Low
**Mental ill-health in general (no specified mental health condition) – individual-level social capital**									
De Silva et al., (2005)	Systematic Review	UK, Scotland, USA, Russia, South Africa, Zambia	14^b^	Adults with common mental disorders (depression and anxiety)	1992–2003	Narrative Synthesis	Social Capital	Common mental disorders (onset and symptoms)	Critically low
Ehsan & De Silva et al., (2015)	Systematic Review	Mexico, USA, UK, Greece, Japan, Australia	33^b^	Adults with common mental disorders (depression and anxiety)	Inception-2014	Narrative Synthesis	Social Capital	Common mental disorders (onset and symptoms)	Low
**COVID-19 context**									
**Covid-19 Perinatal/postnatal depression - social support**									
Fan et al., (2021)	Systematic Review	Israel, Sri Lanka, China, Turkey, Italy, Belgium, Colombia, Japan, the United States, Iran	19	Pregnant women	2020	Narrative Synthesis	Social Support	Psychiatric symptoms of depression and anxiety	Low
**Covid 19: PTSD – social support**									
Hong, Kim and Park, (2021)	Systematic Review	China, Italy, Spain, Israeli, Ireland, USA, Poland	16	All adults	2020	Narrative Synthesis	Social Support	Post-traumatic stress symptoms and post-traumatic stress disorder (PTSS and PTSD), depression, anxiety	Critically low
**Covid: 19: Eating disorders – social isolation**									
Miniati et al., (2021)	Systematic Review	Saudi Arabia, Spain, UK, Italy, Turkey, France, Lebanon, Australia, USA, Canada, Germany	21	Existing eating disorder diagnosis	2020–2021	Narrative Synthesis	Social Isolation	Eating disorders (Anorexia, Bulimia, Binge Eating)	Critically low

^a^ Review paper states that 88 studies were included in the review, but only 70 papers were referenced and authors did not respond to requests for the full list. The types of study included in this umbrella review is derived from the 70 studies that are referenced in the paper.

^B^ Only individual-level social capital studies are included in this umbrella review.

Abbreviations: DSM = Diagnostic and Statistical Manual of Mental Disorders (diagnostic manual); ICD = International Classification of Diseases (diagnostic manual); OCD = obsessive compulsive disorder; SAD = seasonal affective disorder; GAD = generalised anxiety disorder; PTSS = Post-traumatic stress symptoms; PTSD = post-traumatic stress disorder UK = United Kingdom; USA = United States of America

**Table 3:** Summary of Evidence (N = 53 studies)

Author & year	Population		Outcome	Types of studies included	Main Relevant Findings
**Depression – loneliness**					
Cohen-Mansfield et al., (2016)	Older Adults		Depression	11 cross-sectional, 1 longitudinal study	Loneliness was significantly associated with ‘depression’ both cross-sectionally (11 studies) and longitudinally (1 study, which also found a cross-sectional association) (12 relevant studies in total). The definition of depression included studies measuring low mood and uselessness (1 study), lack of taking initiative (1 study) and expecting negative reactions from caregivers (1 study), as well as studies measuring ‘depressive symptoms’ (1 study). What was meant by ‘depression’ in other studies was not specified (8 studies).
Erzen & Çikrikci (2018)	Adults		Depression (unclear if refers to symptom severity or diagnosis)	62 cross-sectional and 8 longitudinal studies	Meta-analysis found loneliness was moderately significantly associated with depression (*r* = 0.5). This relationship held when carers, elderly, students, patients were examined separately.
**Depression – loneliness, social support and social network size**					
Choi et al., (2015)	Older Adults		Depression symptoms	8 cross-sectional studies	Both subjective (e.g., loneliness, perceived social support) and objective (e.g. low social engagement, low social support) types of social isolation were associated with higher depressive symptoms.
Courtin & Knapp et al., (2017)	Older Adults		Depression	18 cross sectional, 8 longitudinal, 3 mixed methods	The authors report that the evidence reviewed ‘clearly showed’ that loneliness is an independent risk factor for depression in old age. The relationship between social isolation and depression was unclear as only three studies included in the review had looked at this, although 2/3 did find some evidence for an association.
Worrall et al., (2020)	Older Adults		Depressive symptom severity	26 cross-sectional and 11 longitudinal studies	Cross-sectional studies suggested that loneliness is associated with depressive symptoms (4/5 studies, remaining 1 study found no significant relationship). ‘Substantial’ evidence (29/36 studies) for social support as a protective factor from depression, with consistent findings across cross-sectional and longitudinal studies (remaining 7 studies found no significant relationship). Findings on the effect of larger social networks varied within and between cross-sectional and longitudinal studies (2/5 studies found protective effect, 3 found no significant relationship).
**Depression – social support and/or social networks**					
Edwards et al., (2020)	Active Christian clergy-members (7.6%United Methodist; 39.2% Catholic; 12.2% Presbyterian; 1% other Protestant denominations)		Association between rates of depression and level of social support among Christian Clergy	3 longitudinal and 10 cross-sectional studies	(1) Increased rates of depression in Christian clergy are associated with perceived levels of social support. (2) A small-to-moderate negative associations between social support and depression, with stronger associations in the two studies using single items or their own questions to measure social support compared to those using standardised measures.
Gariépy et al., (2016)	All ages		Depression (diagnosis or symptoms)	70 cross-sectional and 30 longitudinal studies	For children and adolescents (31 studies), adults (36 studies) and older adults (33 studies), meta-analyses found significant associations between higher social support and lower/absent depression symptoms (pooled OR = 0.2, OR = 0.74 and OR = 0.56 respectively). Sources of support most consistently seem to be protective against depression are: parents, teachers and family in children and adolescents (findings were less consistent for friends); first spousal support, then family, then friends and then children for adults; support from spouses, followed by friends for older adults (support from children showed less consistent findings). Parental and family support was particularly important for girls. Emotional support was most consistently associated with lower depression in adults. Findings were similar for adult men and women, whereas a significant protective association was more consistently found in girls and older men than boys or older women. For children/young people and older adults, estimates were stronger for cross-sectional versus cohort studies.
Guo & Stensland et al., (2018)	Older Chinese and Korean immigrants in USA		Depressive symptoms	30 cross-sectional and 2 longitudinal studies	Eight out of nine included studies reported that low or diminished social support over time was associated with more depressive symptoms. Three studies that examined different types of support found that emotional support was associated with lower depression. Mixed findings regarding whether the size and/or strength of social networks is associated with depression: about half reported a negative relationship and half found no significant association. Living arrangements, frequency of kin/non-kin contact, and positive family relations were not consistently related to depression, but negative family interactions were.
Guruge et al., (2015)	Immigrant women and youth		Depressive symptoms	6 cross-sectional and 6 longitudinal, 22 qualitative studies	Association between lack of social support and depression among immigrant women was well supported (including by 5 longitudinal quantitative studies), although one longitudinal study failed to find a significant association. Poor social support, including from their spouse, was identified as one of the key risk factors for postpartum depression in immigrant women (2 longitudinal, 1 qualitative).
Hall et al., (2018)	LGBQ adolescents (15–24 years of age)		Depression (symptom severity or clinical threshold)	3 cross-sectional and 4 longitudinal studies	5/6 cross-sectional studies found that participants with greater support from friends and/or family (in one case measured as family closeness and contact with friends) experienced lower depression symptoms. A further 3 papers report on the same longitudinal study and 2 of these found cross-sectional and longitudinal negative associations between social support from friends and/or family and depressive symptoms (although the review itself does not clearly delineate cross-sectional and longitudinal associations). A fourth longitudinal paper using a different sample found that depression was negatively associated with social support at T2 (although not clear from the reporting whether this is over time).
Mohd et al., (2019)	Older adults in Asia		Depression (self-reported or diagnosed)	18 cross-sectional and 6 longitudinal studies	Eleven cross-sectional studies (8 rated as good quality) found that low social support was significantly associated with higher depressive symptoms. Higher satisfaction with social support significantly associated with lower depression symptoms in 2/3 studies (1 prospective cohort). Support from family (3 studies, of which 1 was a prospective cohort design) and friends (1 study) was found to reduce depressive symptoms. Emotional support was associated with reduced depression symptoms in six studies including 3 prospective cohort studies. 5/12 cross-sectional studies found good perceived social support was associated with fewer depressive symptoms. Significant association between having a larger size of network and fewer depressive symptoms (2 prospective cohort studies, 1 cross-sectional). A larger social network composed of mostly family members was associated with reduced rate of depression compared with having friends (1 prospective cohort study, 1 cross-sectional).
Qiu et al., (2020)	Chinese adults > age 55		Risk factors for Depressive symptoms	4 cross-sectional studies in meta-analysis involving social support	Fair or good social support was found to be a protective factor against onset of depression (OR = 0.94, 95% CI: 0.84–0.97).
Rueger et al., (2016)	Childhood and Adolescence (Ages under 20)		Depression (diagnosis, symptoms)	293 cross-sectional and 48 longitudinal studies	Meta-analysis found social support was significantly moderately associated with depression (*r* = 0.26), with a particularly strong effect size for available (i.e., perceived to be there if needed) versus enacted support. This significant association held for different sources of support: family, teacher, general peer, and close friend, but associations were larger for support from family and the general peer group (then teachers, then close friends). No gender differences were found. The association between peer social support and depression was stronger for children and younger adolescents than for older adolescents. The relationship between family social support and depression was consistent across all ages.
Santini et al., (2015)	Adults from general population		Depression (symptoms or diagnosis)	28 cross-sectional and 22 longitudinal studies	The strongest and most consistent findings were significant negative associations between depression and perceived emotional support, perceived instrumental support, and large, diverse social networks. Perceived emotional support (PES) significantly negatively associated with depressive symptoms in 32/35 studies: 17 were cross-sectional studies and 14 of were prospective studies (2 low quality, 7 moderate and 5 good) and found that higher levels of PES were protective against depression, whilst lower levels were associated with presence/onset/development of depression. Two of the 3 studies that failed to find a significant association were prospective studies of moderate quality. Similar negative associations were found for 8/12 studies for received emotional support (5 prospective) and 11/12 studies of perceived instrumental support (3 prospective). All 3 studies that looked at both perceived and received social support found the former was more strongly associated with depression (2 prospective). Findings for received instrumental support were mixed: 2/10 studies found a negative relationship, 3/10 found a positive relationship and 4/10 not finding a significant association and 1 prospective study found received emotional and instrumental support predicted symptom deterioration only in people with depression at baseline. 5/8 studies found emotional support to more strongly associated with depression than instrumental (2 prospective of high quality) whereas 3 studies concluded the opposite (1 prospective moderate). Social support from friends was equally important in terms of predicting depression as family support (5/7 studies, 1 prospective) although 2 prospective studies found that only family had an effect. Large social networks were found to be protective against depression in 9/13 studies (5 prospective, high quality) whereas 4 studies found no significant association (2 prospective). Four cross-sectional studies found a significant negative relationship between network diversity and depression outcomes 9/12 studies found an association between living alone or without a partner positively associated with depression (4 prospective).
Schwarzbach et al., (2014)	Older adults		Depression (dimensional diagnosis, prevalence, incidence)	10 cross-sectional and 7 longitudinal studies	Cross-sectional studies found social support (7/9 studies), emotional support (4/7 studies) and relationship quality (5/5 studies) were negatively associated with depression symptoms. These associations were supported by longitudinal studies: social support (3/4 studies), received emotional support (2/3 studies) and satisfaction with social support (2/2 studies) were associated with lower depression. 4/6 cross-sectional studies suggested larger and more diverse networks were associated with lower depression symptoms, and this was supported by 2 longitudinal studies.
Visentini et al., (2018)	Patients with chronic depression		Depression diagnosis	5 cross-sectional, 5 case-control, 7 longitudinal, 1 qualitative studies	Patients with chronic depression rated their perceived social support significantly lower than those in the healthy population (4/6 studies; 1 study found no difference comparing women with dysthymia to those without a history of mood disorder; 1 study found fewer friends before onset of depression in the patient group compared to healthy controls but no difference in perception). 6/8 studies found chronic depression was associated with significantly lower perceived social support compared to individuals with non-chronic depression disorders or who had recovered and remitted. One study found no significant difference in social support to ‘count on’ between those with chronic depression and who had recovered. Social networks of patients with chronic depression appeared to be smaller than those of healthy individuals, patients with non-chronic major depression and other disorders.
**Perinatal/postnatal depression & Anxiety - social support**					
Bayrampour et al., (2018)	Pregnant women		Anxiety (symptoms or diagnosis)	10 cross-sectional, 12 longitudinal/prospective studies	Of 14 studies that were rated as of moderate or strong quality, no studies failed to find a bivariate association, 9 studies found a multivariate negative association between social support and antenatal anxiety, but 2 studies failed to do so.
Bedaso et al., (2021)	Pregnant mothers (18 years +)		Any diagnosed depressive disorders and general anxiety disorder according to ICD and DSM or depressive disorders and general anxiety disorder based on the valid screening tool	37 cross-sectional, 26 longitudinal studies	Low social support found to have a significant positive association with antenatal depression AOR: 1.18 (95% CI: 1.01, 1.41) based on 45 studies, 57% of which were cross sectional. 37 of 45 studies reported a significant positive correlation. In relation to antenatal anxiety, AOR: 1.97 (95% CI: 1.34, 2.92) based on 9 studies, 8 of which were cross sectional and 6 included longitudinal analysis. Narrative conclusions were consistent with this. 15 studies in depression, 12 reported a significant relationship (correlation or association) and of the 8 anxiety ones, 7 reported a significant relationship.
Desta et al., (2021)	Postpartum women with postpartum depression in Ethiopia		Prevalence of post-partum depression	4 cross-sectional studies	Poor social support was a common predictor that significantly associated with increased risk of postpartum depression [POR = 6.27 (95%CI: 4.83, 8.13)] among postpartum women.
Nisar et al., (2020)	Chinese women in perinatal period		Onset of perinatal depression	10 cross-sectional studies	(1) The prevalence of perinatal depression in Chinese women was negatively associated with economic status and social support. (2) Social support before and after childbirth was a strong protective factor for perinatal depression.
Qi et al., (2021)	Chinese women who have given birth to at least one child including those living in countries other than China		Risk factors for the onset of post-partum depression	4 case control, 5 cohort studies	General agreement with prior studies that social support can be a protective factor against PPD (OR 2.57; 95% CI 2.32–2.85) but one of the weaker findings across wider evaluation of risk factors.
Razurel et al., (2013)	Peri- and Post-natal Mothers		Periand Post-natal depression symptoms	9 cross-sectional, 14 longitudinal/prospective, 1 randomised controlled trial RCT, 1 survey design studies	Nine longitudinal studies found lower scores for postnatal social support were related to higher scores of postnatal depressive symptoms. Negative correlation between satisfaction with family social support and postpartum depressive symptoms (3 cross-sectional studies). Three longitudinal studies found that social support from the partner seems to be a protective factor against postpartum depressive symptoms.
Tarsuslu et al., (2020)	Fathers aged 30–36 on average in post-partum period (< 1 year.)		Post-partum depression measured on differing scales but established ones. No further information regarding severity or onset given	3 cross-sectional, 2 cohort studies (studies relevant to our research question)	Social support cited as one of risk factors for PPD, with five of included studies relevant to this. This was judged to be one of stronger impacting factors (age, economic status, ethnicity alongside it) but clear comparison of importance could not be made.
Tolossa et al., (2020)	Ethiopian women in post-partum period		Prevalence of postpartum depression	5 cross-sectional studies	All five studies showed a significant association between social support and PPD. Pooled results showed PPD 6.5x higher among women who lacked social support (95% CI 2.59, 16.77).
Zeleke et al., (2021)	Mothers in post-partum period (< 1 year.)		Prevalence of postpartum depression	3 cross-sectional studies	Poor social support gave increased odds (OR = 3.57;95% CI[2.29–5.54]) of developing PPD.
**Depression, anxiety, and OCD – loneliness and social isolation or social support or social capital**					
Gilmour et al., (2020)	70% Young adults		Mental health conditions with specific reference to depression and anxiety.	5 cross-sectional studies	(1) Facebook-based social support was found to predict better general mental health. (2) Social support drawn from Facebook was predictive of lower levels of depression, depressive mood, and symptomology. (3) Within the high socially anxious group, Facebook-based social support significantly predicted greater psychological well-being, whereas face-to-face social support did not. Within the low socially anxious group, face-to-face social support significantly predicted greater psychological well-being; however, Facebook-based social support had no significant relationship with psychological well-being. (No statistics available.)
Loades et al., (2020)	Children & Adolescents		Depression & anxiety symptom severity	44 cross-sectional and 19 longitudinal studies	45 cross-sectional studies examined the relationship between depressive symptoms and loneliness and/or social isolation: ‘most’ reported moderate to large correlations (*r* = 0.12–0.81) and 2 studies found lonely individuals were 5.8–40 times more like to score above clinical cut-offs for depression. 12/15 longitudinal studies found loneliness explained a significant amount of the variance in severity of depression symptoms several months to several years later. In 1 study, duration of peer loneliness not intensity was associated with depression 8 years later (from age 5 to 13); in contrast, family related loneliness was not independently associated with subsequent depression. 23 cross-sectional studies examined symptoms of anxiety and found small to moderate associations with loneliness/social isolation (*r* = 0.18–0.54); 1 study using odds ratios found loneliness was associated with increased odds of being anxious of 1.63–5.49 times. Two studies found duration of loneliness was more strongly associated with anxiety than intensity of loneliness. Social anxiety (*r* = 0.33–0.72) and generalized anxiety (*r* = 0.37–0.4) were associated with loneliness/social isolation (2 cross-sectional studies). 3/4 studies assessing the longitudinal effect of loneliness on anxiety found loneliness was associated with later anxiety (2 related to social anxiety specifically). Cross-sectional studies also found associations between social isolation/loneliness and panic (1 study), suicidal ideation (3 studies), self-harm (1 study) and disordered eating (1 study). One longitudinal study found internalising symptoms were associated with prior loneliness in primary-school-age children whereas another study found no association between adolescent suicidal ideation and prior loneliness.
Mahon et al., (2006)	Adolescents (11–23 years of age)		Depression (unclear if refers to symptoms, severity or diagnosis)	30 (no information on breakdown but mostly cross sectional)	Meta-analysis found significant positive relationship between depression and loneliness (*r* ~ 0.6) was found from 33 hypotheses derived from 30 studies. Meta-analysis found social anxiety was significantly positively associated with loneliness with a moderate effect size (*r* ~ 0.4; r = 0.35 when outliers were removed) (investigated via 15 hypotheses derived from 12 studies).
**Anxiety and ADHD - loneliness**					
Hards et al., (2021)	Predominantly children or adolescents with heightened distress, mental health problems, or diagnoses based on internationally recognized (DSM or ICD)		Depression, anxiety, ASD and ADHD	8 cross-sectional studies	Seven studies examined the cross-sectional relationship between loneliness and severity of mental health symptomstr, four of which examined loneliness and social anxiety. Three studies reported socially anxious children as significantly lonelier than those not anxious with small to moderate effect sizes (0.31). Another two studies showed positive correlations between loneliness and severity of symptoms. For autistic participants, there were moderate association between anxiety and loneliness in both younger adolescents (< 14 years) (*r* = -0.33) and older adolescents (*r*=-0.44).
**Anxiety – social support and/or social network**					
Zimmermann et al., (2020)	Adults in general population who had a clinically diagnosed anxiety disorder. They looked at anxiety disorders across the spectrum (outcomes included OCD, Specific Phobia, SAD (social anxiety disorder), GAD (generalised anxiety disorder etc.)		Anxiety (prevalence)	4 longitudinal studies	Heterogenous findings but overall, a lack of social contact was not a risk factor but loneliness did show evidence of being one. In relation to underlying studies: social support was not a risk factor for SAD when adjusted for subthreshold SAD at baseline (1 study). In another study low social support was associated with x4 the risk factor for SAD when controlling for age and gender. In a third study after adjusting for age, income and current disease limited social contact did not present itself as a risk but perceived loneliness was a risk factor for any anxiety disorder. Coping skills, including social support, were found to be protective in the development of specific phobias (1 study).
**PTSD – social support**					
Allen et al., (2021)	Children and Adolescents (6–18 years of age)		Overall relationship between social support and PTSD; relationship between severity of PTSD and degree of social support	46 cross-sectional and 4 prospective/longitudinal studies	The current review found a weak correlation between social support (support from peers, family and teachers) and PTSD (*r* = -0.12, 95% CI -0.16 to -0.07, k = 41) in children and young people following trauma (War, Abuse, Hurricane, Community violence, Flood, Tornado, Cancer, Tsunami, Earthquake, Terrorist attack, Typhoon) with the strongest effect size for social support that were provided by teachers (*r* = -0.20, 95% CI, -0.15 to -0.24, k = 5); however, the effect size is still considered small.
Blais et al., (2021)	Service members or veterans in the U.S. military		Severity of PTSD symptoms	38 cross-sectional studies	The types of social support (e.g., perceived, enacted, structural) did not moderate the association between PTSD and social support; Lower levels of social support were associated with more severe PTSD symptoms: *r* = − 0.33 (95% CI:[− 0.38, − 0.27], Z = − 10.19, p < 0.001).
Tirone et al., (2021)	Adult betrayal trauma survivors		PTSD symptom severity	29 cross-sectional studies	Overall weighted effect size was small to medium (*r* = − 0.25) suggesting higher levels of positive support and lower levels of negative support were associated with lower PTSD symptom severity. Substantial degree of heterogeneity. Studies focusing on absence of social support reported a larger effect size than those reporting on positive presence of social support.
Trickey et al., (2012)	Children and Adolescents (6–18 years of age)		Post- traumatic stress disorder (PTSD), both diagnosis and symptom severity	4 (no information on breakdown)	A medium-to-large effect size was observed for a significant positive relationship between PTSD symptoms and low social support ${\hat{\rho}}$ = 0.33 (95% CI [0.13, 0.53]).
Zalta et al., (2021)	All participants had to be exposed to a DSM-5 criterion event and had to be > 18 years; treatment studies were excluded and study population chosen on basis of their PTSD were excluded		PTSD symptom severity	150 cross-sectional and longitudinal studies	Higher levels of social support were associated with lower PTSD symptom severity. Reporting cross sectional studies, longitudinal studies respectively, type of social support was a significant predictor of effect size: negative social reactions (*r*=-0.40 & *r*=-0.41); perceived support (*r*=-0.27 & *r*=-0.22); structural support (*r*=-0.19 & *r*=-0.21)) and enacted support (*r*=-0.15).
**PTSD and Depression – social support and/or social network**					
Scott et al., (2020)	Adults 18 years plus following bereavement after sudden / violent death		Clinical diagnosis of PTSD or depression and symptom severity of the same	9 cross-sectional; 1 longitudinal studies	For depression: limited evidence that social support associated with reduced risk of clinical diagnosis; 4 studies reported positive association depression and reduced social support and two reported a partial association, 1 with limited association and 1 (poor quality) with strong correlation. PTSD: 6 studies reported partial association (nothing to suggest reduced symptom severity or less likely to meet level for clinical diagnosis), and 4 with positive association.
**Psychosis – loneliness**					
Chau et al., (2019)	Adults with and without clinical diagnosis of psychosis		Psychosis (symptoms, psychotic experiences)	26 cross-sectional, 3 longitudinal, and 2 experimental design studies	Meta-analysis found a medium association between loneliness and positive psychotic experiences (*r* = 0.302; 30 studies: 3 longitudinal, 1 experimental, 26 cross-sectional) and paranoia (*r* = 0.448).There was a medium association between loneliness and negative psychotic experiences across 15 cross-sectional studies (*r* = 0.347).The associations between loneliness and both positive and negative psychotic experiences were significantly smaller among clinical (positive: *r *= 0.149; negative: 0.127) than non-clinical samples (positive: *r* = 0.389; negative: *r* =0.479).
Michalska da Rocha et al., (2018)	People diagnosed with a psychotic disorder		Psychosis (symptoms)	11 cross-sectional and 2 longitudinal studies	Meta-analysis found a moderate significant positive cross-sectional association between psychosis symptom severity and loneliness (*r *= 0.32) from 13 studies assessed as being of moderate quality. Although 2 longitudinal studies were included, the authors used cross-sectional baseline data or averages across timepoints.
Lim et al., (2018)	People diagnosed with a psychotic disorder		Psychosis (diagnosis)	9 cross-sectional studies	Individuals with psychosis were found to be significantly lonelier than control participants in the general population (2 studies). One study found that both negative and positive symptoms correlated significantly with loneliness (and one study found that state anxiety partially mediates the relationship between anxiety and paranoia) but two studies failed to find a significant relationship between loneliness and psychotic symptom severity; a fourth study did not find a difference in loneliness between people diagnosed with schizophrenia with or without auditory hallucinations. Within people with psychosis, loneliness seems to be significantly associated with depression symptoms (4/6 studies).
**Psychosis – social networks and/or social support**					
Degnan et al., (2018)	Adults with schizophrenia		Schizophrenia (symptomatic and/or functional outcome)	13 cross-sectional and 3 longitudinal studies	Meta-analytic pooled effect sizes found that smaller social network size was significantly moderately associated with more severe overall psychiatric symptoms (4/5 cross-sectional studies found significant associations, Hedge’s *g*=-0.53) and negative symptoms (7/8 studies including 1 longitudinal RCT, which was not included in the meta-analysis due to insufficient data, found a significant association; Hedge’s *g*=-0.75), but not positive symptoms (7 studies: 3 found significant cross-sectional negative associations but 6 other studies did not, and these included 3 longitudinal studies, 2 of which were not included in the meta-analysis due to insufficient data) or social functioning (3 cross-sectional studies including 100% schizophrenia samples). Narrative synthesis (which included three RCTs identified in the review) suggested that larger network size was associated with improved global functioning, but findings for affective symptoms and quality of life were mixed. 2 longitudinal RCTs reported significant cross-sectional but not temporal associations between more social contacts and greater global functioning. 3/5 cross-sectional studies found a positive association between number of social contacts and subjective quality of life.
Gayer-Anderson and Morgan (2013)	Adults, first episode psychosis & general population samples with psychotic experiences or schizotypal traits		Early episode psychosis (first episode)	36 cross-sectional studies	11 studies (3 longitudinal) compared network size (various measures including total size and number of particular relationships, e.g., family, friends, confidants), frequency of contact or perceived level or adequacy of social support between samples of individuals with first episode psychosis and various comparison groups. All but one study, which compared number of friends in adolescence and frequency of contact 1-year pre-contact between Finnish versus Spanish cases, found at least one significant difference between social network size/structure/contact or social support: people with first episode psychosis were generally found to have smaller networks and less perceived and less satisfactory social support. There is some evidence that differences in network size are specifically in number of and contact with friends, with individuals with a first episode having significantly fewer friends than controls (3 studies, 2 longitudinal). There was also evidence from 3 studies (1 longitudinal) that people with first episode psychosis have fewer confidents than comparison groups. There were inconsistent findings in relation to whether duration of untreated psychosis and various social network measures (8 studies, 5 longitudinal). There was some support of associations between measures relating to social support and psychosis symptoms in general population samples (9/11 studies, 1 longitudinal).
Palumbo et al., (2015)	Adults (≥ 18 years of age) with psychotic disorder		Psychosis (diagnosis)	16 cross-sectional and 4 longitudinal studies	Across included studies, patients with psychosis had on average 11.7 individuals in their social networks (range 4.6–44.9; 20 studies), while the average number of friends was 3.4 (range 1–5; 7 studies). Social networks were family-dominated with on average 43.1% of network members being relatives and 26.5% of members being ‘friends’ (14 studies). Higher levels of negative symptoms may be associated with smaller networks in individuals with psychosis (2 studies). No significant associations were found between social network size and age of onset/ length of prodromal period (1 study), or illness duration (2 studies).
**Bipolar Disorder – social support**					
Greenberg et al., (2014)	Adults with bipolar disorder		Bipolar disorder (general term “bipolar disorder”, manic or depressive episodes)	4 cross-sectional and 11 longitudinal studies	Individuals with bipolar disorder experience a lower level of social support than controls but the level of social support is similar to that of patients with other psychiatric diagnoses (5 cross-sectional studies). Negative cross-sectional associations were found between depressive symptoms (2 studies) or manic episodes (1 study). Findings from 10 longitudinal studies were mixed: social support has been found to influence manic or depressive episode relapse (4 studies) or only depressive relapse (4 studies) or manic relapse (1 study) or has not been found to affect relapse (1 study).
Studart et al., (2015)	People with bipolar disorder		Bipolar disorder (general term “bipolar disorder”, symptoms, mania and depression)	5 cross-sectional and 8 longitudinal studies	12/13 studies (8 cohort) found associations between social support and bipolar symptoms, recovery or recurrence. Patients with bipolar disorder had low social support (1 cross-sectional) including when compared to controls or a community sample (3 cross-sectional studies). Low social support was associated with higher risk of relapse (2 cohort study), recurrence of manic and depressive episodes (1 cohort study, 1 cross-sectional) or just depressive episodes (2 cohort studies). Higher social support was found to be associated with quicker recovery from depressive episodes (1 cohort study). One 6-month cohort study found stronger treatment alliances were associated with higher patient social support. 1 cohort study with a 2-year follow up did not find a significant association between social support and onset or recovery.
**Depression, Bi-Polar, Psychosis & Anxiety Disorders – loneliness & perceived social support**					
Wang et al., (2018)	Adults with mental illness		Depression, schizophrenia, bipolar disorder and anxiety disorders (relapse, measures of functioning or recovery, symptom severity, global outcome)	34 longitudinal studies	Prospective studies provide substantial evidence that people with depression who have poorer perceived social support have worse symptoms (11/13 studies, *r* = 0.10–0.61), recovery (6/7 studies) and functioning outcomes (2/5 studies). Some preliminary evidence was found for associations between perceived social support and outcomes in schizophrenia (2/2 studies but did not adjust for baseline scores), bipolar disorder (4/4 studies: lower perceived social support was consistently found to significantly predict greater depression, more impaired functioning and longer time to recovery; however, findings were inconsistent regarding severity of manic symptoms: 1/3 studies found perceived social support predicted more severe manic symptoms at follow-up). Significant associations between social support at baseline and outcomes at follow up for people with anxiety disorders (3/3 studies). One study found that lower perceived social support was predictive of more severe anxiety and depressive symptoms later on. Another found that higher perceived social support predicted greater remission rates at 6-month follow-up. A third study in older adults with Generalised Anxiety Disorder found an association between greater perceived social support at baseline and greater average quality of life over time (although without adjustment for baseline scores). Two studies looked at mixed samples with various mental health problems: one found that greater loneliness at baseline predicted more severe depression 1 year later controlling for baseline depression severity; the other study found greater perceived social support significantly predicted higher subjective quality of life 18 months later in people with severe mental illness but did not control for baseline levels of quality of life.
**Eating disorder – social support**					
Arcelus et al., (2013)	Patients with eating disorders & non-clinical populations (university students) and controls		Eating disorder diagnosis	4 cross-sectional studies	3/3 studies found significant associations between social support and eating disorder diagnosis. Individuals (3/4 studies were female only) with bulimia nervosa were found to have lower perceived social support from family and friends and more negative interactions (1 study), to have fewer strategies for seeking social support in response to stressful situations, controlling for anxiety and depression (1 study), and to both have fewer people to provide emotional support and be less satisfied with the quality of emotional support from relatives (1 study), compared to healthy controls. Individuals with eating disorders were found to have less structural social support and those with anorexia or bulimia were found to have less emotional or practical support compared to controls; those with anorexia were less like to identify a spouse/partner as a support figure compared to those with bulimia (1 study).
**Mental ill-health in general (no specified mental health condition) – social support**					
Casale & Wild, (2013)	Adult caregivers who have HIV and adult caregivers caring for HIV/AIDS affected children		Psychological distress, depression, anxiety, psychiatric disorder symptoms	16 cross-sectional and 1 longitudinal studies	Significant positive association between social support and mental health outcomes such as lower psychological distress or depressive symptoms (7 studies) but 2 studies found a negative relationship between social support and mental health outcomes: 1 study found receiving more social support was significantly related to higher depressive symptoms in low-income mothers with late stage HIV/AIDS and another study found that although support from neighbours/friends was associated with lower psychological distress, greater emotional support from children was associated with greater psychological distress. 1 study found that greater emotional closeness or attachment in relationships was associated with lower anxiety among HIV-positive mothers of young children.
Tajvar et al., (2013)	Older people in Middle Eastern countries		Mental health	9 cross-sectional studies	8/9 studies found an inverse association between social support and poor mental health, although 3 studies did not control for potential confounders. There were consistent associations between perceived social support and mental health but not for received/available support (2 studies).
**Mental ill-health in general (no specified mental health condition) – individual-level social capital**					
De Silva et al., (2005)	Adults with mental illness (common mental disorder, anxiety or depression)		Mental illness (diagnosis, symptoms, onset, recovery, time to recovery, incidence rates, death rate from suicide)	14 cross-sectional studies	14 studies measured individual-level social capital. Evidence for an inverse relation between cognitive social capital and common mental disorders was found (7/11 effect estimates). There was some evidence for an inverse relation between cognitive social capital and child mental illness (2/7 effect estimates), and between combined measures of social capital and common mental disorders (2/2 studies). Findings on associations between structural social capital and common mental disorder were mixed: 3/11 found an inverse association, 7/11 did not find a significant association, and 1/11 found a positive association.
Ehsan & De Silva et al., (2015)	Adults with common mental disorders		Common mental disorders (risk of the disorder)	27 cross-sectional and 6 longitudinal studies	High individual level cognitive social capital was associated with reduced risk of developing common mental health disorders (5/5 cohort studies), and this was supported by 27/33 cross-sectional effect estimates. Findings from 5 cohort studies were mixed for structural social capital (3/5 found a significant effect); cross-sectional findings were also mixed (11/25 effect sizes indicated an inverse relationship, 3/25 indicated a positive relationship).
**COVID-19 context**					
**PTSD – loneliness and social support**					
Hong, Kim and Park, (2021)	All adults		Onset and severity of symptoms of PTSD	16 cross-sectional studies	Two studies found loneliness was the strongest predictor of PTSD. Three studies found that social support was associated with a decreased risk of impaired mental health such as anxiety, depression and PTSD. One study showed that social support from family was associated with decreased risk of depression and PTSS, whereas support from friends or partners was not associated with mental health.
**Perinatal/postnatal depression - social support**					
Fan et al., (2021)	Pregnant adult women		Likelihood of onset of depression or anxiety	19 cross-sectional studies	Pregnant women were more concerned about others than themselves during Covid-19, and younger pregnant women seem to be more prone to anxiety, while social support can reduce the likelihood of anxiety and depression developing.
**Eating disorders – social isolation**					
Miniati et al., (2021)	Adults with Eating disorders (Anorexia, Bulimia, Binge Eating)	Eating disorder severity (all eating disorders)		17 cross-sectional, 2 qualitative, and 2 longitudinal cohort studies	Social isolation was related to the exacerbation of symptoms in patients with EDs who were home-confined with family members (No statistics available).

Abbreviations: RCT = randomised controlled trial; ED = eating disorders; PTSD/PTSS = post-traumatic stress disorder/syndrome; OCD = obsessive–compulsive disorder; UK = United Kingdom; USA = United States of America; PES = Perceived emotional support, PPD = postpartum depression

In total, 53 systematic reviews were included in the final umbrella review (Tables 2 and 3; Fig. 1), which together included 1,657 studies, of which 340 (21%) were longitudinal (Supplementary Table S3, showing study methodologies). Supplementary Table S4 provides details of the 147 studies that were included in more than one review: thus fewer than 10% of primary studies were included in more than one review. Of the 53 included reviews, 31 used narrative synthesis, 17 included meta-analyses, and five conducted both meta-analysis and narrative synthesis. Locations of studies included within the systematic reviews encompassed Europe, Asia, Africa, North and Central America and Australasia, although there were very few studies from lower income countries. Reviews were published between 2005 and 2021, with only three published prior to 2013. No deviations from the PROSPERO protocol were identified.

Twenty-one studies focused on depression, of which 16 related solely to depression and the other four to depression in conjunction with other disorders such as anxiety, obsessive compulsive disorder (OCD), or psychosis. Ten reviews investigated peri- or post-natal depression; seven psychosis; six Post-Traumatic Stress Disorder (PTSD); four mental health in general (i.e. not differentiating conditions); one anxiety and a further two anxiety in conjunction with other disorders. Three reviews focused specifically on mental health and loneliness during the COVID-19 pandemic.

Five different social constructs featured as the analysed exposures across the reviews: 29 reviews focused on social support; 11 on loneliness, sometimes together with measures of social isolation, and 2 on social capital. Eleven reviews investigated a combination of these constructs alongside social networks. Clear differentiations were not always made between constructs such as loneliness and social isolation, so that sometimes we report on these together below. See Table 2 for more detail.

The quality of reviews according to AMSTAR ratings tended to be low (Supplementary Table S2): 27 reviews were assessed as critically low in quality, 14 as low quality, and 12 as of moderate quality. Common critical flaws were failure to establish clear review methods pre-publication, such as via PROSPERO pre-registration, and insufficient discussion by review authors of the risk of bias when interpreting results. Of note only two reviews included evidence from randomised controlled trials (RCTs): one review included three RCTs alongside other study designs but could only summarise findings in its narrative review as they were not eligible for inclusion in their meta-analysis [26], whilst one other review included one RCT alongside other study designs [27].

Methodological and statistical heterogeneity across reviews prevented useful meta-analyses so we undertook a narrative synthesis. Headings below reflect topics on which there is evidence to report: we do not include sections for combinations of social constructs and mental health outcomes for which we found no relevant reviews.

### Depression

Twenty-one reviews focused on links between depression and a range of constructs, sometimes including more than one social outcome in each review.

#### Loneliness and depression

Four reviews focused on older adults [28–31], one on adults in the general population [32], one on adults experiencing mental illness [33], and two on adolescents [10, 34]. Reviews varied in operationalisations of “depression” (clinical diagnosis versus symptoms), but findings appeared similar regardless of the approach to measurement.

For example, a recent narrative systematic review reported that both cross-sectional and longitudinal studies found statistically significant associations between loneliness and/or social isolation and depressive symptoms in children and adolescents, with longitudinal effects found several years later, and cross-sectional effect sizes being moderate to large [10]. An earlier meta-analysis also found a significant positive relationship between loneliness and depression across 30 studies in adolescents and young people aged 11–23 years [34]. A meta-analysis of mostly cross-sectional studies across the adult age range reported that loneliness was moderately positively correlated with depression, including in distinct models for carers, the elderly, students and service users [32]. The only systematic review focusing solely on longitudinal studies among adults with mental health conditions found only a single study investigating the longitudinal relationship between loneliness and outcomes for people with depression, which reported loneliness to be a predictor of worse depression outcomes [33].

Loneliness was also found to be positively associated with depressive symptoms in older adults. Three reviews of primarily cross-sectional studies reported links between loneliness and perceived social support, and loneliness and depressive symptoms in most included studies [28, 29, 31]. A scoping review using systematic methods included cross-sectional and longitudinal studies, and reported that the overall evidence ‘clearly showed’ a link between loneliness and depression [30].

#### Social support and depression

Eighteen reviews investigated depression and social support (Tables 2 and 3). Seven focused on older adults [28, 30, 31, 35–38], four on adults of all ages [39–42], and five on children and adolescents [10, 43–46], including one on LGBQ young people [45]. Wang et al. [33] investigated the relationship between perceived social support and outcome in people with depression, and Edwards et al. [47] reviewed literature on social support and depression among Christian clergymen, a group at high risk of depression.

Three reviews included meta-analyses, all reporting inverse relationships between social support and depressive symptom severity and/or diagnosis. A review including cross-sectional and longitudinal studies found associations between more social support and fewer depressive symptoms for children and adolescents, adults, and older adults [40]. A meta-analysis of studies investigating social support and depression among children and adolescents also found a moderate effect, which held across different sources of support [44]. A meta-analysis of cross-sectional studies in older adults in China reached a similar conclusion [38].

Results varied in other more population-specific reviews. For example, in a narrative synthesis investigating depression amongst adults experiencing traumatic bereavement [41], there was some evidence that social support was associated with depression diagnosis or symptom outcome, but no such evidence in the only included longitudinal study. A systematic review of longitudinal studies in adults experiencing mental illness found that for people with depression, lower perceived social support at baseline was predictive of worse symptoms in 11/13 studies, reduced recovery in 6/7 studies and poorer functioning outcomes in 2/5 studies at subsequent follow-up assessments [33].

In the general population, less perceived social support was associated with depression in a review focused on adolescents [44], and another focused on adults [39]. Likewise, three reviews based primarily on cross-sectional studies examined the relationship between satisfaction with social support and depressive symptom severity and/or risk of developing depression and found an inverse relationship [27, 35, 37].

Most reviews did not differentiate between sources of social support. However, for children and young people, one review found that social support from peers was especially important for children and younger adolescents [44]. A second review of mainly cross-sectional studies [40] found that support from parents was more strongly associated with depression risk than support from friends, particularly for girls. There were inconsistent findings for adults regarding which sources of support are most significant in terms of associations with depression [39, 40]. Among older adults, spousal support had the strongest association with lower levels of depression, followed by support from friends, with less evidence for support from children [40].

#### Social networks and depression

Six reviews explored associations between social network size and depression onset, diagnosis and/or symptom severity. Four focused on older adults [31, 35–37], one on adults in the general population [39] and one on adults with chronic depression [48].

In one review of moderate quality, large social networks were found in most studies to be protective against the onset of depression in the general population (9/13 studies, including five prospective studies of high quality) whereas four studies (two prospective) found no statistically significant association [39]. For people already experiencing chronic depression, social networks appeared to be smaller than those of healthy individuals, or of patients with non-chronic major depression and other disorders [48].

Findings on the association between social network size and depressive symptoms were mixed, with reviews identifying some studies suggesting a protective effect of large social networks and others finding no relationship [36, 37].

Two reviews reported on associations with social network composition in older adults, with one reporting a social network composed mainly of family rather than friends to be associated with lower rates of depression in older adults [37], while a second review reported fewer depressive symptoms among those with a larger and more diverse network [35].

### Peri-natal Conditions

#### Social Support and peri-natal depression

Ten reviews looked at peri- or post-natal depression and its relationship to social support in countries including China, the USA, Australia and Ethiopia [22, 27, 49–56].

Peri-natal findings were consistent in suggesting that lower social support was associated with elevated odds of developing depression. In a meta-analysis of 45 studies, 26 of which were cross-sectional, 37 found a significant negative relationship between social support and developing depression. The narrative element of the paper, which drew on 15 studies, reported similar findings [55]. Two earlier systematic reviews drew similar conclusions. A review of 10 cross-sectional, 14 longitudinal and one RCT studies found lower social support was related to higher scores for both peri- and post-natal depressive symptoms [27]. The review also suggested that social support may play a mediating role between peri- and post-natal symptoms. In a meta-analysis of 10 cross-sectional studies focusing on Chinese women [50], the conclusions were similar to those above, although the economic status of women tended to matter more than social support. In a review of risk factors for paternal peri-natal depression, five out of seventeen studies (three cross sectional, two longitudinal) identified lower social support as a risk factor alongside age, economic status and ethnicity [56] (Tarsuslu et al. 2020).

#### Social support and peri-natal anxiety

Two reviews investigated peri-natal anxiety [22, 55]. All nine studies included in a meta-analysis reported a significant negative relationship between social support and developing peri-natal anxiety [55]. A second review similarly reported lower levels of social support to be a risk factor for ante-natal anxiety in most included studies [22].

### Anxiety

Five reviews investigated social constructs and anxiety: three related to loneliness [10, 34, 57] and two to social support [33, 58].

#### Loneliness and anxiety

Three reviews investigated loneliness and anxiety, all focused on children and adolescents. A rapid systematic review on children and adolescents in the general population found small to moderate associations between loneliness and social anxiety in cross-sectional studies, while three out of four longitudinal studies found that loneliness was associated with greater subsequent rates of anxiety [10]. An earlier meta-analysis, also focused on adolescents, found mostly significant positive associations between loneliness and social anxiety with a moderate effect size [34].

A recent rapid systematic review [57] regarding children and adolescents with established mental health conditions (including depression, anxiety, and neurodevelopmental disorders) also identified associations between loneliness and anxiety (especially social anxiety) in most included studies, including among autistic participants.

#### Social support and anxiety

The reviews investigating social support and anxiety focused on the adult population. A recent review [58] reported mixed evidence as to whether social support was a protective factor against severity of anxiety and, on balance, concluded it was not. A systematic review including only longitudinal studies of people with mental health conditions at baseline found significant associations between perceived social support and subsequent anxiety outcomes [33].

### Psychosis

Seven systematic reviews investigated social relationship constructs and psychosis. Four focused on loneliness and/or perceived social support [13, 14, 23, 33], and three on social network size and composition [26, 59, 60].

#### Loneliness and/or perceived social support and psychosis

The only review focusing solely on longitudinal studies found no relevant studies on loneliness in relation to psychosis outcomes. It did report preliminary evidence from two studies for positive associations between perceived social support, and life satisfaction and social functioning [33].

Most of the studies included in the three other reviews were cross-sectional (Table 3). Two included meta-analyses indicating moderate positive associations between loneliness and psychotic symptom severity [13, 23]. Chau et al. [23] distinguished different types of psychosis symptoms and found associations between loneliness and both positive symptoms (30 studies, only three of which were longitudinal), and negative symptoms (15 cross-sectional studies).

The third systematic review that focused on loneliness in psychosis included perceived social isolation in the search terms and reported inconsistent findings, with some studies reporting associations between loneliness and psychosis symptoms and others finding none [14].

#### Social networks and psychosis

Three systematic reviews investigated social networks among people with diagnosed psychotic disorders [26, 59, 60].

Degnan et al. [26] conducted a meta-analysis of cross-sectional data and found that smaller social network size was moderately associated with more severe overall psychiatric symptoms and negative symptoms, but not positive symptoms. Most of the studies had samples of participants with a diagnosis of schizophrenia. In one narrative review, participants with first episode psychosis were found to have smaller social networks and reported less satisfactory social support compared to general population controls [60]. In the same review, scores on measures related to psychosis or schizotypy were found to be negatively related to social network size and social support in samples from the general population who reported psychotic experiences or had high levels of schizotypy traits [60].

Another narrative review found evidence that a larger proportion of the social networks of people with psychosis are family members rather than friends, although lack of general population control groups and inconsistent methods for measuring social networks limited conclusions [59].

### PTSD

#### Social support and PTSD

Seven reviews examined the relationship between PTSD and social support. Two of these focused on children [61, 62], two reported on the adult population in general [42, 63], and three on specific adult populations including military veterans [64]; betrayal trauma survivors [65]; and adults bereaved by sudden or violent death [41]. All papers focused on symptom severity and most included studies were cross-sectional.

In a series of meta-analyses by the same group of authors [63–65], the association between social support and symptom severity was investigated across three different populations: military veterans, adults in the general population, and adult betrayal survivors. In each population, a moderate association was found between greater social support, perceived or enacted, and less severe PTSD symptoms.

All six studies in the review on bereaved adults found that greater social support was associated with lower likelihood of meeting the threshold for PTSD and/or less severe PTSD symptoms [41], including one longitudinal study.

Two meta-analyses investigated PTSD and social support in children and adolescents [61, 62]. A meta-analysis of four cross-sectional studies found a significant positive association between low social support and PTSD with a medium-to-large effect size [62]. A more recent meta-analysis found only a small effect size between social support and PTSD symptoms, with considerable heterogeneity across results [61].

#### Bipolar disorder and constructs related to social relationships

Three systematic reviews examined relationships between social support and bipolar disorder [21, 33, 66]. Overall, the reviews found that people with bipolar disorder have lower levels of social support than the general population [21, 66]. Longitudinal data from one review did not support a clear association between social support and bipolar episode relapse (either manic or depressive) [21]. However, the other two reviews did find evidence to suggest that people with bipolar disorder who have greater social support have fewer recurrences overall and fewer depressive episodes [33, 66]. One review including solely longitudinal studies found that lower perceived support was a significant predictor of greater depressive symptom severity and a longer time to recovery in people diagnosed with bipolar disorder [33].

#### Eating disorders and constructs related to social relationships

Two systematic reviews investigated eating disorders and a social relationships construct. One looked at social isolation during the Covid-19 pandemic [67] (see COVID-19 section below). Another review focused on social support in relation to anorexia nervosa and bulimia nervosa: all four included studies were cross-sectional [20]. People with eating disorders were found to report receiving less emotional and/or practical social support than general population controls.

#### General mental health

Five reviews included studies not focused on specific diagnoses, including two examining relationships between social support and mental health in general [68, 69], and a third at social support in relation to all mental health disorders, including groups with a mixture of diagnoses [33]. A further two reviews looked at individual-level social capital in relation to common mental health disorders [70, 71].

#### Social support and mental ill-health

A systematic review on adults caring for children with HIV reported mixed findings from cross-sectional studies on the relationship between social support and mental ill-health [68]. A second review investigated the mental health of older people in Middle Eastern countries and found that higher levels of social support, especially perceived social support, were associated with better mental health outcomes in eight out of nine studies. In their review of studies investigating the longitudinal relationships between mental health problems and both loneliness and perceived social support, Wang et al. [33] reported on two studies with samples of people with a mixture of mental health conditions. One study found that loneliness at baseline predicted subsequent depression and the other that greater perceived social support predicted higher subsequent quality of life.

#### Social capital and mental health

Two narrative systematic reviews using largely cross-sectional data (Table 3) found an inverse association between individual-level cognitive social capital and common mental disorders [70, 71], apparent in both cross-sectional and longitudinal studies in the latter review. Individual-level cognitive social capital was defined by the authors as quality of social interactions, measured by asking about participation in social relationships and perceptions of the quality of those relationships. Findings regarding structural social capital (which aims to measure the quantity of social interactions) and common psychiatric conditions were a mixture of positive associations, negative associations and no associations. See Table 3 for details.

#### COVID-19 pandemic

There were three reviews investigating the association between social constructs and mental ill health during the COVID-19 pandemic. One focused on loneliness [67], one on social support [49] and another on both loneliness and social support [42].

A review focusing on loneliness and eating disorder symptom severity [67] reported mixed and low-quality evidence as to whether social isolation policies during the pandemic were associated with greater eating disorder symptoms, with no clear overall conclusions about the impact of home confinement during the pandemic on eating disorder symptoms. A review focusing on loneliness in relation to PTSD during the pandemic [42] found loneliness to be the strongest psychological predictor of PTSD, with the authors noting both the prevalence and distressing nature of loneliness related to social distancing and isolation measures during Covid-19. In the same review, there was consistent evidence in six cross sectional studies suggesting people with adequate social support were less likely to have PTSD. In one of those studies, the type of social support was important: family support, but not support from friends or partners, was associated with lower PTSD prevalence.

A systematic-review of cross-sectional studies focused on pregnant women’s experiences during the Covid-19 pandemic concluded that women who received more social support were less likely to develop depression or anxiety [49], although associations with socio-economic variables were stronger.

## Discussion

### Main findings

This umbrella review summarises a body of evidence linking various social constructs relevant to social relationships with a range of mental health diagnoses, with depression and psychosis the conditions most commonly investigated. Our review includes all the constructs identified as related to subjective and objective aspects of social relationships in an influential conceptual review [19] and all major mental health conditions, offering a broad stock-take of the current state of evidence in this field as reflected by systematic reviews.

Regarding what is known so far, much more literature has focused on depression than on any other condition. However, even for this condition, much of the literature reported in reviews is cross-sectional and limited in shedding light on mechanisms or the role of particular forms of social support and social relationships. Regarding cross-sectional findings, most measures of constructs related to loneliness and social isolation appear associated with depression in the expected directions. Some systematic reviews on social support and social network size identify some longitudinal evidence suggesting a protective effect against depression onset or against more severe symptoms. A systematic review focused on adolescents found that loneliness predicts onset of depression and of anxiety.

Regarding other conditions, various reviews report cross-sectional associations between poorer social support and onset and/or severity of symptoms in anxiety (including perinatal anxiety) and PTSD, with some longitudinal evidence for relationships between social support and onset or severity of anxiety. For the relationship between psychosis and loneliness, the two reviews that include a meta-analysis of cross-sectional data both concluded that there is an association between loneliness and psychosis symptoms. However, another review concluded the evidence was of insufficient quality for such a conclusion to be firmly drawn and did not attempt a meta-analysis. A psychosis diagnosis was also found to be cross-sectionally associated with more limited social network size. Some reviews of the longitudinal relationship between social indicators and bipolar disorder reported social support as protective against relapse. Finally, a single review regarding the relationship between eating disorders and social support found cross-sectional evidence for less social support among people with eating disorders.

Many gaps emerged from this overview of reviews. Even for depression, which has yielded the largest amount of literature, reviews report only a limited body of longitudinal evidence to allow clear conclusions to be reached about temporal relationships and potential causality. Most reviews pool studies across countries and cultures, even though relevant social indicators and their impacts might be expected to vary cross-culturally. Likewise, whether patterns vary by gender, demographic group and other socio-demographic factors cannot generally be extrapolated from the reviews. Most measures of social constructs were relatively lacking in nuance: global indicators of loneliness tended to be reported rather than differentiating sub-types, although some studies made distinctions between different types of social support (for example from friends versus family). Evidence on support from peers with relevant personal experience of mental health problems was also generally not reported in the syntheses but has considerable potential practical relevance.

Reviews of literature on anxiety, bipolar and eating disorders identified relatively few studies, most cross-sectional. While a somewhat greater number of primary studies was retrieved in the reviews on psychosis (including schizophrenia), high quality longitudinal studies from which an understanding of causality can be achieved were not retrieved. No review reporting on associations with “personality disorder” diagnosis was found, despite the centrality of social relationships to the difficulties many people with such a diagnosis face, nor were reviews found regarding specific anxiety-related conditions such as obsessive compulsive disorder and social anxiety. Social support was the most commonly assessed social construct in reviews and there were no reviews looking at relationships between individual-level social capital and specific mental health disorders. Where reviews were found, they tended to be of low or critically low quality, confirming significant further potential for conducting high quality reviews in this sphere.

### Strengths and Limitations

The main strength of this review was the aggregation of evidence regarding the relationships between multiple constructs related to social connection/relations and a range of mental health conditions in both clinical and general population samples. We followed PRISMA guidelines, pre-registered the protocol and used independent screening to improve the rigor of the review process. Grouping of studies was informed by clinician team members’ views as to what would be more clinically meaningful.

The main limitation of this umbrella review was the generally low quality of included reviews, according to our team’s quality appraisals. Most meta-analyses and reviews included in this umbrella review used cross-sectional data so causality could not be inferred. Because we aggregated the findings of systematic reviews rather than primary studies, details of primary studies not reported in specific systematic reviews will have been lost. Publication dates varied, and primary studies published since review searches will not have been captured in this review of reviews. Some reviews were also included in multiple systematic reviews, although this applied to fewer than 10% of included primary studies. Further limitations of our approach, reflecting the wide scope of our review, were that we double screened all studies only at full text stage, and did not search grey literature (we anticipated yield in terms of good quality unpublished systematic reviews would be low), consult experts to seek further references or carry out hand-searching of reference lists. Our search strategy was reviewed by multiple experienced reviewers within our team (BLE, AP, SJ), but not subject to external peer review. We screened five databases, including both comprehensive and subject-specific ones, as well as repositories of systematic reviews, but we did not include Google Scholar, CENTRAL, or SCOPUS, databases which are included in recent Cochrane Collaboration guidance on designing comprehensive searches for individual studies [72], and which might have retrieved additional systematic reviews. We also did not search for grey literature: this is not recommended in guidance on umbrella reviews that we followed [24] or in the Cochrane Collaboration recommendations for an Overview of Systematic Reviews [73]: however, it may have resulted in the omission of systematic reviews not published in peer-reviewed sources, for example if produced by policy bodies.

The broad scope of our view across many mental health conditions and constructs related to social relationships, and variations in how these were measured, mean that it is challenging to make direct comparisons between findings of different reviews, and a metasynthesis was not feasible. Thus while we can summarise conclusions from a substantial number of reviews, we cannot present statistically robust summary findings for main research questions, nor was grading of overall strength of evidence feasible. Even though we included a wide range of mental health conditions and symptoms, a significant limitation is that in the interests of feasibility, we did not include well-being or positive conceptualisations of mental health, advocated as important for holistic and recovery-focused understanding of mental health [74].

### Clinical, research and policy implications

Some of the many gaps in the evidence as reported by systematic reviews have already been identified above, especially in relation to conditions other than depression, to longitudinal studies, and to better quality reviews. However, there is consistent evidence to support associations between certain social constructs and specific psychiatric conditions, particularly depression, post-natal mental health conditions and psychosis, and thus a clear rationale for conducting further longitudinal studies to understand outcomes and the mechanisms underpinning associations. Hypotheses to drive mechanistic studies can be derived not only from cross-sectional studies but also from qualitative research (e.g., Birken et al. [75]) and from relevant lived experience.

Established associations between social constructs and mental health conditions suggest potential benefits in raising clinician awareness of these potential influences on mental health. Work to embed measurement of loneliness and other constructs related to social isolation in routine clinical examinations might yield benefits in raising clinicians’ awareness of their influence on mental health and the potential scope for helpful social interventions. Documenting repeated measures of loneliness could also facilitate longitudinal analyses of anonymised data from electronic health records, advancing understanding of prevalence and potential mechanisms and identifying potential targets for intervention. The UK government recommends using the 3-item UCLA Loneliness Scale and a single item direct measure of loneliness [76]. This presents a low question burden for clinicians and patients as well as being a valid and reliable measure [77]. Cohort studies that include both good quality measures of loneliness, social support and related constructs, and substantial numbers of participants with diagnoses such as psychosis, bipolar disorder, “personality disorder”, anxiety disorders or eating disorders would be of considerable value.

Whilst better epidemiological evidence is very desirable, action to alleviate loneliness among people with mental health problems should not need to wait until a comprehensive body of high-quality observational evidence is available. Especially regarding depression, the substantial evidence for a close relationship with social constructs suggests that the development and testing of theory-driven interventions to prevent onset or improve prognosis by pathways involving social construct is justified, especially as interventions with social targets tend to draw strong support from service users [78]. Regarding other conditions, we are still further from a clear understanding of causal relationships, and high-quality research elucidating pathways between social relationships and conditions such as eating disorders, psychosis, bipolar and “personality disorders” is still needed to develop interventions and policy initiatives with secure foundations. However, given the overall evidence that loneliness and social isolation have negative effects on a wide range of aspects of quality of life and health, including a substantial impact on physical health, there is a case for attempting to alleviate these difficulties among people with mental health conditions that are associated with a greater risk of being lonely and/or lacking in support.

### Lived experience commentary by Beverley Chipp

Loneliness and social isolation both have correlations with mental health, however they are distinct entities, and each may exist without the other, or co-occur. Not differentiating, or even using the terms interchangeably, has clouded good research and is challenging to unpick within systematic reviews. The second challenge is the generalised grouping of mental health conditions. This welcomed paper is one of the few which looks at specific diagnoses.

More research is needed beyond depression, for being lonely may itself generate that. The paucity of systematic reviews for anxiety and PTSD is unfortunate as both conditions can compel people to withdraw from social contact. In PTSD it may be a necessary stage in order to decompress, feel safe and heal before restorative socialisation can commence. Anxiety, conversely, may require timely habituation and exposure therapy. These nuances, learned from our lived experience community, highlight the importance of examining discrete mental health conditions, and whilst we can confidently say that building social capital is good, what type is most beneficial or needed for each cohort remains underexplored.

Overall, the relationship between both loneliness and social isolation and mental health conditions and symptoms is shown to be strong, but qualitative work reveals that the relationship is bidirectional [75]. This may extend too to some of the associated behaviours, such as eating disorders, or self-harming. In these ‘chicken or egg’ situations, which is it better to treat first? Or both simultaneously? Again, this may differ according to primary diagnosis. There is a need for more focused longitudinal studies exploring causality, and for co-produced qualitative research to gain deeper understanding of the dynamics. However, the sheer weight of the evidence already suggests there should be no delays in incorporating routine screening for social isolation and loneliness into practice, and establishing appropriate social programmes at the earliest opportunity, particularly noting the protective effect against relapse. Understanding social isolation’s knock-on costs in the bigger picture would make this a prudent investment.

Beverley Chipp (November 2022).

### Electronic supplementary material

Below is the link to the electronic supplementary material.

Supplementary Material 1: **Tables S1-S4** for social relationships & mental health umbrella review

## Data Availability

This review relied on published papers and these are available via journal websites, which in some case require institutional access. All the data analysis reported in the review is provided in tables in the main manuscript or in the supplementary material.

## Abbreviations

DSM: Diagnostic and Statistical Manual of Mental Disorders (diagnostic manual)
ICD: International Classification of Diseases (diagnostic manual)
OCD: obsessive compulsive disorder
SAD: seasonal affective disorder
GAD: generalised anxiety disorder
PTSS: Post-traumatic stress symptoms
PTSD: post-traumatic stress disorder UK = United Kingdom
USA: United States of America
