# Supplementary material for: Associations between constructs related to social relationships and mental health conditions and symptoms: an umbrella review
Source: BMC Psychiatry. 2023 Sep 4;23:652. doi: 10.1186/s12888-023-05069-0 (PMC10478264; doi:10.1186/s12888-023-05069-0)
Supplement: Supplementary file 1 — Supplementary Material 1: Tables S1-S4 for social relationships & mental health umbrella review [file 12888_2023_5069_MOESM1_ESM.docx]

Supplementary Table S1: Example Search Strategy (for Medline)

Search terms below were used in Medline and adapted for the other databases searched

Terms Mapped to Subject Heading and unmapped

| search |  |
| --- | --- |
| 1 | Exp loneliness/ |
| 2. | Lonel*.mp. |
| 3. | Exp social isolation or social isolation.mp. |
| 4. | Social network*.mp. |
| 5. | Social support.mp. or exp social support/ |
| 6. | Confiding.mp. |
| 7. | Confide.mp. |
| 8. | Social contact*.mp. |
| 9. | social relation*.mp. |
| 10. | Social capital.mp. or exp social capital/ |
| 11. | Mental health/ or mental disorders/ OR mental.mp. |
| 12. | Psychiatry*.mp. or exp psychiatry/ |
| 13. | Exp psychotic disorders/ or exp schizophrenia OR schizo*.mp. |
| 14. | Psychosis.mp. |
| 15. | Exp depression or depress*.mp. |
| 16. | Mani*.mp. or mania.mp. |
| 17. | Exp bipolar or manic.mp. |
| 18. | Exp anxiety/ or anxiety.mp. or exp anxiety disorders/ |
| 19. | Exp “feeding and eating disorders”/ |
| 20. | Eating disorder.mp. |
| 21. | Anorexia nerovosa.mp. or anorexia nervosa/ |
| 22. | Bulimia nervosa.mp. or bulimia nervosa/ |
| 23. | Binge eating disorder.mp. or binge eating disorder/ |
| 24. | personality disorders or personality disorder.mp. or borderline personality disorder/ |
| 25. | Borderline personality disorder.mp. |
| 26. | Emotionally unstable personality.mp. |
| 27. | Histrionic personality disorder or histrionic personsality.mp. |
| 28. | Narcissistic personality.mp. |
| 29. | Antisocial personality.mp. or antisocial personality disorder/ |
| 30. | Paranoid personality.mp. or Paranoid personality disorder/ |
| 31. | Schizoid personality.mp. or Schizoid personality disorder/ |
| 32. | Schizotypal personality disorder/ or Schizotypal personality.mp. |
| 33. | Avoidant personality.mp. |
| 34. | Dependent personality disorder/ or dependent personality.mp. |
| 35. | Obsessive compulsive personality.mp. or compulsive personality disorder/ |
| 36. | 1 or 2 or 3 or 4 or 5 or 6 or 7 or 8 or 9 or 10 |
| 37. | 11 or 12 or 13 or 14 or 15 or 16 or 17 or 18 or 19 or 20 or 21 or 22 or 23 or 24 or 25 or 26 or 27 or 28 or 29 or 30 or 31 or 32 or 33 or 34 or 25 |
| 38. | “systematic review”/ or “review”/ or review.mp. |
| 39. | Meta analysis.mp. or meta analysis/ |
| 40. | 38 or 39 |
| 41. | 36 and 37 and 40 |

**Supplementary Material Table S2: AMSTAR quality ratings**

| **Study** | **PICO** | **Review methods established in advance** | **Comprehensive search strategy** | **Duplicate study selection** | **Duplication extraction** | **List of excluded studies** | **Details for including studies** | **Assessed risk of bias (RoB)** | **Meta-analysis methods (if performed)** | **Assessed RoB in meta-analysis (if performed)** | **RoB factored into interpretation** | **Discussed heterogeneity** | **Publication bias assessed (if quantitative analysis)** | **Rating of overall confidence in the results of the review** |
| --- | --- | --- | --- | --- | --- | --- | --- | --- | --- | --- | --- | --- | --- | --- |
| Allen et al., (2021) | Yes | Yes | Yes | No | No | No (nc) | Yes | Partial Yes | Yes | Yes | Yes | Yes | Yes | Moderate |
| Arcelus et al., (2013) | Yes | No | Partial Yes | No | No | No (c) | Partial Yes | Partial Yes | N/A | N/A | Yes | N/A | N/A | Low |
| Bayrampour et al., (2018) | Yes | No | Partial Yes | Yes | Yes | No (nc) | Yes | Partial Yes | N/A | N/A | Yes | Yes | N/A | Low |
| Bedaso et al., (2020) | Yes | Yes | Partial Yes | Yes | Yes | No (nc) | Yes | Partial Yes | Yes | Yes | Yes | Yes | Yes | Moderate |
| Blais et al., (2021) | Yes | Yes | Yes | Yes | Yes | No (nc) | Partial Yes | Yes | Yes | Yes | Yes | Yes | Yes | Moderate |
| Casale & Wild (2013) | Yes | No | Partial Yes | No | No | No (c) | Partial Yes | No | N/A | N/A | No | No | N/A | Critically low |
| Chau et al., (2019) | Yes | Partial Yes | Partial Yes | No | No | No (nc) | Yes | Yes | Yes | Yes | No | Yes | Yes | Low |
| Choi, Irwin & Cho (2015) | Yes | No | No | Yes | Yes | No (nc) | Partial Yes | No | N/A | N/A | No | Yes | N/A | Critically low |
| Cohen-Mansfield et al. (2017) | Yes | No | Partial Yes | No | No | No (c) | Partial Yes | No | N/A | N/A | No | No | No | Critically low |
| Courtin & Knapp, (2017) | Yes | No | Partial Yes | No | No | NO (nc) | Partial Yes | No | N/A | N/A | No | No | No | Critically low |
| De Silva et al., (2005) | No | No | Partial Yes | No | No | No (nc) | Yes | No | N/A | N/A | No | No | N/A | Critically low |
| Degnan et al., (2018) | Yes | Yes | Partial Yes | Yes | Yes | No (nc) | Yes | Partial Yes | Yes | Yes | Yes | Yes | Yes | Moderate |
| Desta et al., (2021) | Yes | No | Partial Yes | Yes | Yes | No (c) | No | Partial Yes | No | Yes | Yes | Yes | Yes | Critically low |
| Edwards et al., (2020) | Yes | No | Partial Yes | No | No | No (nc) | Partial Yes | Partial Yes | N/A | N/A | N/A | N/A | No | Critically low |
| Ehsan & De Silva, (2015) | Yes | No | Partial Yes | Yes | No | No (nc) | Yes | Yes | N/A | N/A | Yes | Yes | N/A | Low |
| Erzen & Cikrikci, (2018) | No | No | Partial Yes | No | No | No (c) | No | No | Yes | No | No | No | Yes | Critically Low |
| Fan et al., (2021) | Yes | No | Partial Yes | No | No | No (c) | Partial Yes | Partial Yes | Yes | No | Yes | Yes | Yes | Low |
| Gariepy et al., (2016) | Yes | No | Partial Yes | Yes | Yes | No (c) | No | Yes | Yes | Yes | No | Yes | No | Critically Low |
| Gayer-Anderson & Morgan, (2013) | No | No | Partial Yes | No | No | No (c) | Partial Yes | No | N/A | N/A | No | No (very limited) | N/A | Critically Low |
| Gilmour et al., (2020) | Yes | No | Yes | Yes | Yes | No (nc) | Yes | Yes | N/A | N/A | Yes | N/A | N/A | Low |
| Greenberg et al., (2014) | Yes | No | Partial Yes | No | No | No (nc) | Partial Yes | No | N/A | N/A | No | No | N/A | Critically Low |
| Guo & Stensland, (2018) | Yes | No | Partial Yes | Yes | Yes | No (c) | Partial Yes | No | N/A | N/A | No | No | N/A | Critically Low |
| Guruge et al, (2015)* | Yes | No | Partial Yes | No | No | No (c) | Yes | No | N/A | N/A | No | No | N/A | Critically Low |
| Hall (2018) | Yes | Yes | Partial Yes | Yes | Yes | No (nc) | Yes | No | N/A | N/A | No | No | N/A | Critically Low |
| Hards et al., (2021) | Yes | No | Partial Yes | Yes | Yes | No (nc) | Partial Yes | Yes | N/A | N/A | Yes | N/A | N/A | Low |
| Hong, Kim & Park, (2021) | Yes | No | Partial Yes | Yes | Yes | No (nc) | Partial Yes | Yes | N/A | N/A | No | No | N/A | Critically low |
| Lim et al., (2018) | Yes | No | Partial Yes | Yes | Yes | No (nc) | Partial Yes | Partial Yes | N/A | N/A | Yes | Yes | N/A | Moderate |
| Loades et al., (2020) | Yes | No | Partial Yes | Yes | Yes | No (nc) | Partial Yes | Partial Yes | N/A | N/A | Yes | No | N/A | Moderate |
| Mahon et al., (2006) | Yes | No | No | Yes | Yes | No (nc) | No | Yes | No | No | No | Yes | No | Critically Low |
| Michalska da Rocha et al.,(2018) | Yes | Yes | Partial Yes | Yes | No | No (nc) | Yes | Yes | Yes | Yes | Yes | Yes | Yes | Moderate |
| Miniati et al., (2021) | Yes | No | No | Yes | Yes | No (nc) | Partial Yes | No | N/A | N/A | No | N/A | N/A | Critically low |
| Mohd et al., (2019) | Yes | Yes | Partial Yes | No | Yes | No (nc) | Yes | No | N/A | N/A | No | Yes | N/A | Critically Low |
| Nisar et al., (2020) | Yes | Yes | Partial Yes | Yes | Yes | No (nc) | Partial Yes | Yes | Yes | Yes | Yes | Yes | Yes | Moderate |
| Palumbo et al., (2015) | Yes | No | Partial Yes | No | Yes | No (nc) | Yes | No | N/A | N/A | No | Yes | N/A | Critically Low |
| Qi et al., (2021) | Yes | No | Partial Yes | Yes | Yes | No (nc) | Yes | No | Yes | No | No | Yes | Yes | Critically Low |
| Qiu et al., (2020) | Yes | No | No | Yes | Yes | No (c) | No | No | Yes | No | No | Yes | No | Critically Low |
| Razurel et al., (2013) | No | No | Partial Yes | Yes | Yes | No (nc) | Yes | No | N/A | N/A | No | No | N/A | Critically Low |
| Rueger et al., (2016) | Yes | No | Partial Yes | Yes | Yes | No (nc) | Partial Yes | Yes | Yes | Yes | Yes | Yes | Yes | Low |
| Santini et al., (2015) | No | No | Partial Yes | Yes | No | No (nc) | Partial Yes | Partial Yes | N/A | N/A | Yes | Yes | N/A | Low |
| Schwarzbach et al., (2014) | Yes | No | Partial Yes | No | No | No (nc) | Yes | Partial Yes | N/A | N/A | No | Yes | N/A | Critically Low |
| Scott et al., (2020) | Yes | Yes | Partial Yes | Yes | Yes | No (nc) | Yes | Yes | N/A | N/A | Yes | Yes | N/A | Moderate |
| Studart et al., (2015) | No | No | Partial Yes | No | No | No (nc) | Yes | No | N/A | N/A | No | No | N/A | Critically Low |
| Tajvar et al., (2013) | No | No | Partial Yes | No | No | No (nc) | Yes | Partial Yes | N/A | N/A | Yes | No | N/A | Low |
| Tarsuslu et al., (2020) | No | No | Partial Yes | No | No | No | Partial Yes | No | N/A | N/A | No | No | N/A | Critically Low |
| Tirone et al., (2021) | Yes | Yes | Yes | Yes | Yes | No (nc) | No | No | Yes | No | No | Yes | Yes | Critically Low |
| Tolossa et al., (2020) | Yes | No | Partial Yes | Yes | Yes | No (c) | Yes | Partial Yes | Yes | No | Yes | Yes | Yes | Low |
| Trickey et al.,(2012) | Yes | No | Partial Yes | Yes | Yes | No (c) | Yes | Yes | Yes | Yes | Yes | Yes | Yes | Critically Low |
| Visentini et al., (2018) | Yes | Yes | Partial Yes | Yes | Yes | No (nc) | Yes | Partial Yes | N/A | N/A | No | No | N/A | Low |
| Wang et al., (2018) | Yes | Yes | Partial Yes | Yes | Yes | No (nc) | Partial Yes | Partial Yes | N/A | N/A | Yes | Yes | N/A | Moderate |
| Worrall et al., (2020) | Yes | No | Partial Yes | Yes | No | No (nc) | Partial Yes | Partial Yes | N/A | N/A | Yes | Yes | N/A | Low |
| Zalta et al., (2021) | Yes | Yes | Yes | Yes | Yes | No (nc) | Yes | Partial Yes | Yes | Yes | Yes | Yes | Yes | Moderate |
| \| Zeleke et al. (2021) \| \| --- \| | Yes | Yes | Partial Yes | Yes | Yes | No (c) | Yes | Partial Yes | Yes | Yes | Yes | No | Yes | Low |
| \| Zimmermann et al. (2020) \| \| --- \| | Yes | Yes | Partial Yes | Yes | Yes | No (nc) | Yes | Partial Yes | N/A | N/A | Yes | Yes | Yes | Moderate |

*Rapid Reviews or Scoping reviews where the criteria for methodological quality are less robust. For consistency, we have used the same criteria.

Studies are scored based on Amstar guidance (<https://amstar.ca/Amstar-2.php>). In our scoring we sought to keep the wider question in focus: whether the systematic review provides an accurate and comprehensive summary of the results of the available studies. Question 6 asks for a list of reasons for excluding studies. When studies report some explanation but not the full list, we rated this a ‘No’ but not as critically flawed **No (nc).** When studies failed to report any explanation for excluding studies, we rated this as being critically flawed ‘**No (c)’**. No study reported a full list of excluded studies.

**Table S3: Study designs employed by empirical studies included in the eligible reviews**

| **Study Type** | **Total Number of studies** |
| --- | --- |
| Cross-sectional study (some studies presented an analysis of cross-sectional data from a specific wave of a longitudinal cohort study) | 1060 |
| Cohort/ Longitudinal study | 340 |
| Qualitative study | 25 |
| Case-control study | 10 |
| Experimental study | 2 |
| Pilot Randomised Controlled Trial | 1 |
| No information on study design | 219 |
| **Total number of studies** | **1657** |
| **Total studies included in multiple reviews** | **147** |

*Total number of studies includes repeated studies within multiple reviews*

**Table S4: Studies that were included in more than one of the systematic reviews included in the umbrella review**

| **Author** | **Year** | **Type of Study** | **Population** | **Exposure** | **Outcome** | **Included in** |  |  |  |  |  |
| --- | --- | --- | --- | --- | --- | --- | --- | --- | --- | --- | --- |
| **Depression – loneliness (or loneliness and social isolation)** | | | | | | |  |  |  |  |  |
| Adams et al., (2004) | 2004 | Cross-sectional | Older adults aged 60-98 years | Loneliness | Depressive symptoms | Choi et al 2015  Cohen-Mansfield et al, 2016  Courtin et al, 2017  Erzen & Cikrikci, 2018 |  |  |  |  |  |
| Aylaz et al., (2012) | 2012 | Cross-sectional | Adults aged 60 years and over | Loneliness | Depressive symptoms | Choi et al 2015  Erzen & Cikrikci, 2018 |  |  |  |  |  |
| Beeson (2003) | 2003 | Cross-sectional | Spousal caregivers of those with Alzheimer’s disease versus non- caregiving spouses. | Loneliness | Depressive symptoms | Erzen & Çikrikci, 2018  Courtin et al, 2017 |  |  |  |  |  |
| Bisschop et al., (2004) | 2004 | Prospective studies | Adults aged 55–85 years old | Loneliness | Depressive symptoms | Santini et al, 2015  Courtin et al, 2017 |  |  |  |  |  |
| Cacioppo et al., (2010) | 2010 | Longitudinal & Cross-sectional | Older adults (age range not specified) | Loneliness | Depression | Santini et al, 2015  Cohen-Mansfield et al, 2016  Courtin et al, 2017 |  |  |  |  |  |
| Cacioppo et al., (2006) | 2006 | Longitudinal & Cross-sectional | Older adults | Loneliness | Depression | Gariepy et al, 2016  Courtin et al, 2017 |  |  |  |  |  |
| Kara & Mirici (2004) | 2004 | Cross-sectional | Patients with chronic obstructive pulmonary disease and their spouses | Loneliness | Depressive symptoms | Tajvar et al., 2014  Erzen & Çikrikci., 2018 |  |  |  |  |  |
| Lau et al., (1999) | 1999 | Cross-sectional | Children & adolescents | Loneliness | Depressive symptoms | Erzen & Çikrikci, 2018  Schwarzbach et al, 2014 |  |  |  |  |  |
| Luo et al., (2012) | 2012 | Longitudinal | Older Adults | Loneliness | Depressive symptoms | Cohen-Mansfield et al. 2016  Courtin et al, 2017 |  |  |  |  |  |
| Mahon et al., (2001) | 2001 | Cross-sectional | Adolescents (12 – 14 years of age) | Loneliness | Depressive symptoms | Loades et al, 2020  Rueger et al., 2016 |  |  |  |  |  |
| Matthews et al., (2016) | 2016 | Cohort | Young adults | Loneliness | Depressive symptoms | Loades et al, 2020  Erzen & Çikrikci, 2018 |  |  |  |  |  |
| Swami et al., (2007) | 2007 | Cross-sectional | Young adults aged 18-24 years of age | Loneliness | Depressive symptoms | Erzen & Çikrikci, 2018  Loades et al, 2020 |  |  |  |  |  |
| Purwono & French (2016) | 2016 | Cross-sectional | Indonesian-Muslim adolescents (13-15 years of age) | Loneliness | Depressive symptoms | Erzen & Çikrikci, 2018  Loades et al, 2020 |  |  |  |  |  |
| Spithoven et al., (2017) | 2017 | Cross-sectional | Adolescent | Loneliness | Depressive symptoms | Loades et al, 2020  Erzen & Çikrikci, 2018 |  |  |  |  |  |
| Switaj et al., (2014) | 2014 | Cross-sectional | Adults with psychotic disorders | Loneliness | Depressive symptoms | Michalska da Rocha, et al 2018  Chau et al 2019 |  |  |  |  |  |
| Theeke et al., (2012) | 2012 | Cross-sectional | Older adults | Loneliness & social isolation | Depressive symptoms | Choi, H., Irwin, M., Cho, H. 2015  Courtin et al, 2017 |  |  |  |  |  |
| **Depression – loneliness and social support** | | | | | | |  |  |  |  |  |
| Alpass et al., (2003) | 2003 | Cross-sectional | Male aged 60 years of age and above | Loneliness & social support | Depressive symptoms | Choi et al 2015  Courtin et al, 2017  Erzen & Cikrikci, 2018 |  |  |  |  |  |
| Han et al., (2007) | 2007 | Cross-sectional | Adults aged 60 years and above | Loneliness & social support | Depression symptoms | Worrall et al, 2020  Guo & Stensland, 2018 |  |  |  |  |  |
| Hudson et al., (2000) | 2000 | Cross-sectional | Adolescent mothers | Loneliness & social support | Depression scores | Loades et al, 2020  Rueger, 2016dd  Erzen & Çikrikci, 2018 |  |  |  |  |  |
| Liu et al., (2016) | 2016 | Cross-sectional | Adults aged 65 years and above | Loneliness & perceived social support | Depressive symptoms | Guo & Stensland, 2018  Erzen & Çikrikci, 2018 |  |  |  |  |  |
| **Depression – social support / social networks / social capital / social self-efficacy** | | | | | | |  |  |  |  |  |
| Alexandrino- Silva et al., (2011) | 2011 | Cross-sectional | Adults aged 60 years of age and above | Social support | Depressive symptoms | Worral et al, 2020  Santini et al, 2015 |  |  |  |  |  |
| Ang & Molhotra (2016) | 2016 | Cross-sectional | Older adults | Social support | Depressive symptoms | Modh et al., 2019  Worrall et al., 2020 |  |  |  |  |  |
| Auerbach et al., (2011) | 2011 | Longitudinal | Adolescents (12 – 18 years of age) | Social support | Depressive symptoms | Gariepy et al., 2016  Rueger et al., 2016 |  |  |  |  |  |
| Chao (2011) | 2011 | Longitudinal | Older adults (over 60 years of age) | Social support | Depressive symptoms | Modh et al., 2019  Schwarzbach et al, 2014 |  |  |  |  |  |
| Chan et al., (2011) | 2011 | Cross-sectional | Adults aged 60 years and above | Social network | Depressive symptoms | Modh et al, 2019  Worrall et al, 2020  Santini et al, 2015 |  |  |  |  |  |
| Choi & Ha et al., (2011) | 2011 | Cross-sectional | Adults aged 57-85 years of age | Social support (spouse / partner) | Depressive symptoms | Gariepy et al, 2016  Santini et al, 2015 |  |  |  |  |  |
| Chou & Chi (2003) | 2003 | Longitudinal | Older adults (over 70 years of age) | Social support | Depressive symptoms | Modh et al., 2019  Schwarzbach et al, 2014 |  |  |  |  |  |
| Colarossi & Eccles (2003) | 2003 | Longitudinal | Adolescents (15 – 18 years of age) | Social support | Depressive symptoms | Gariepy et al., 2016  Rueger et al., 2016 |  |  |  |  |  |
| Cornman et al., (2003) | 2003 | Longitudinal | All ages in general population/ older adults | Social support | Depression | Schwarzbach et al, 2014  Gariepy et al, 2016 |  |  |  |  |  |
| Fiori et al., (2006) | 2006 | Cross-sectional | Middle aged (30-59 years of age) and older adults (60+ years of age) | Social self-efficacy | Depressive symptoms | Gariepy et al, 2016  Santini et al, 2015 |  |  |  |  |  |
| Fukunaga et al., (2012) | 2012 | Cross-sectional | Adults aged 65 years and above | Living alone | Depressive symptoms | Schwarzbach et al, 2014  Worrall et al, 2020 |  |  |  |  |  |
| Galambos et al., (2004) | 2004 | Longitudinal | All ages in general population/ Children & adolescents | Social support | Depression (diagnosis, symptoms) | Gariepy et al, 2016  Rueger, 2016 |  |  |  |  |  |
| Galand & Hospel (2013) | 2013 | Cross-sectional | All ages in general population/ Children & adolescents | Social support | Depression (diagnosis, symptoms) | Gariepy et al, 2016  Rueger, 2016 |  |  |  |  |  |
| Garcia-Pena et al., (2013) | 2013 | Longitudinal | Older adults (over 60 years of age) | Social support | Depressive symptoms | Santini et al., 2015  Worrall et al., 2020 |  |  |  |  |  |
| George et al., (1989) | 1989 | Cohort | Middle aged and elderly adults with major depression | Social network | Depressive symptoms | Wang et al 2018  Visentini et al, 2018 |  |  |  |  |  |
| Glaesmer et al., (2011) | 2011 | Cross-sectional | Adults aged 60-85 years of age | Social support | Depressive symptoms | Schwarzbach et al, 2014  Santini et al, 2015  Gariepy et al, 2016 |  |  |  |  |  |
| Grav et al., (2012) | 2012 | Cross -sectional | Adults aged 20-89 years of age | Perceived social support (emotional and tangible) | Depressive symptoms | Gariepy et al, 2016  Santini et al, 2015 |  |  |  |  |  |
| Heponiemi (2006) | 2006 | Longitudinal | Adolescents and adults (15-30 years of age) | Perceived social support | Depressive symptoms | Gariepy et al, 2016  Santini et al, 2015 |  |  |  |  |  |
| Kim et al., (2013) | 2013 | Cross-sectional | Immigrant seniors aged 65 and above | Social capital | Depressive symptoms | Ehsan & De Silva, 2015  Guo & Stensland, 2018 |  |  |  |  |  |
| Klima & Repetti (2013) | 2013 | Cross-sectional | Children (9 – 12 years of age) | Social support | Depressive symptoms | Gariepy et al., 2016  Rueger et al., 2016 |  |  |  |  |  |
| Koizumi et al., (2005) | 2005 | Longitudinal | Older adults (over 60 years of age) | Social support | Depressive symptoms | Modh et al., 2019  Santini et al., 2015 |  |  |  |  |  |
| Kuchibhatla et al., (2012) | 2012 | Longitudinal | Adults 60 years of age and above | Social network | Depressive symptomology | Worrall et al, 2020  Santini et al, 2015 |  |  |  |  |  |
| Leung et al., (2007) | 2007 | Cross-sectional | Adults 65 years of age and above | Social support | Depression | Schwarzbach et al, 2014  Santini et al, 2015 |  |  |  |  |  |
| Lin et al., (2014) | 2014 | Cross-sectional | Adults 60 years of age and above | Social network | Depressive symptoms | Worrall et al, 2020  Guo & Stensland, 2018 |  |  |  |  |  |
| Marcotte et al., (2002) | 2002 | Cross-sectional | Adolescents | Social support | Depressive symptoms | Gariepy et al., 2016  Rueger et al., 2016 |  |  |  |  |  |
| Murberg (2009) | 2009 | Longitudinal | Adolescents (16 – 18 years of age) | Social support | Depressive symptoms | Gariepy et al., 2016  Rueger et al., 2016 |  |  |  |  |  |
| Park et al., (2013) | 2013 | Cross-sectional | Older adults (over 60 years of age) | Social support | Depressive symptoms | Choi et al., 2014  Courtin et al, 2017  Guo & Strensland., 2018 |  |  |  |  |  |
| Piboon et al., (2009) | 2009 | Cross-sectional | Older adults (over 65 years of age) | Social support | Depressive symptoms | Modh et al., 2019  Worrall et al., 2020 |  |  |  |  |  |
| Piko et al., (2009) | 2009 | Cross-sectional | Adolescents (14 - 20 years of age) | Social support | Depressive symptoms | Gariepy et al., 2016  Rueger et al., 2016 |  |  |  |  |  |
| Rosario et al., (2005) | 2005 | Longitudinal | LBGQ adolescents | Social support | Depressive symptoms | Hall, 2019  Rueger et al., 2016 |  |  |  |  |  |
| Russell and Taylor (2009) | 2009 | Cross-sectional | Adults aged 60 years and above | Social support | Depressive symptoms | Worrall et al, 2020  Santini et al, 2015 |  |  |  |  |  |
| Shin et al., (2008) | 2008 | Longitudinal | Older adults (over 65 years of age) | Social support | Depressive symptoms | Modh et al., 2019  Schwarzberg et al., 2014  Worrall et al., 2020 |  |  |  |  |  |
| Sonnenberg et al., (2013) | 2013 | Cohort | Adults aged 55-85 years of age without depression | Social support | Prevalence of depression | Gariepy et al, 2016  Santini et al, 2015 |  |  |  |  |  |
| Teo et al., (2013) | 2013 | Cohort | Adults (25-75 years of age) without depression | Social support | Depressive symptoms | Santini et al, 2015  Gariepy et al, 2016 |  |  |  |  |  |
| Tiedt (2010) | 2010 | Cross-sectional | Adults 65 years and above | Social Support | Depressive symptoms | Modh et al, 2019  Santini et al, 2015 |  |  |  |  |  |
| Tsai et al., (2005) | 2005 | Cross-sectional | Adults 65 years and above | Social support & social network | Depressive symptoms | Modh et al, 2019  Schwarzbach et al, 2014  Santini et al, 2015 |  |  |  |  |  |
| Tummala-Narra & Sathasivam-Rueckert (2013) | 2013 | Cross-sectional | Adolescents (12 - 18 years of age) | Social support | Depressive symptoms | Gariepy et al., 2016  Rueger et al., 2016 |  |  |  |  |  |
| Yoo et al., (2016) | 2016 | Cross-sectional | Older adults (over 60 years of age) | Social support | Depressive symptoms | Modh et al., 2019  Worrall et al., 2020 |  |  |  |  |  |
| **Perinatal/postnatal depression & anxiety - =social support** | | | | | | |  |  |  |  |  |
| Abadiga (2019) | 2019 | Cross-sectional | Postpartum women | Social support | Postnatal Depressive symptoms | Desta et al, 2021  Zeleke et al, 2021 |  |  |  |  |  |
| Bayrampour et al., (2015) | 2015 | Longitudinal | Postpartum women | Social support | Postnatal Depressive and general anxiety symptoms | Bayrampur et al., 2019  Bedaso et al, 2021 |  |  |  |  |  |
| Glazier et al., (2004) | 2004 | Longitudinal or Cross-sectional | Postpartum women | Social support | Postnatal depression | Razurel et al, 2019  Bedaso et al, 2021 |  |  |  |  |  |
| Li et al., (2017) | 2017 | Longitudinal | Postpartum women | Social support | Postnatal Depressive and general anxiety symptoms | Bedaso et al, 2021  Qi et al. 2021 |  |  |  |  |  |
| Martini et al., (2015) | 2015 | Longitudinal | Pregnant women | Social support | Anxiety (symptoms, generalised anxiety, overall anxiety disorders in pregnancy | Bayrampur et al., 2018  Bedaso et al, 2021 |  |  |  |  |  |
| Shitu et al., (2019) | 2019 | Cross-sectional | Postpartum women | Social support | Postnatal Depressive symptoms | Desta et al, 2021  Zeleke et al, 2021 |  |  |  |  |  |
| **Depression & anxiety – social support** | | | | | | |  |  |  |  |  |
| La Greca & Harrison (2005) | 2005 | Cross-sectional | Adolescents (14 – 19 years of age) | Social support | Social Anxiety & Depression | Gariepy et al., 2016  Rueger et al., 2016 |  |  |  |  |  |
| Lee et al., (2007) | 2007 | Longitudinal | Women who are pregnant | Social support | Anxiety & depressive symptoms | Bayrampur et al., 2018  Rueger et al., 2016 |  |  |  |  |  |
| **Depression & psychosis - loneliness** | | | | | | |  |  |  |  |  |
| Badock et al., (2015) | 2015 | Cross-sectional | Adults with psychosis | Loneliness | Psychotic and depressive symptoms | Michalska da Rocha, et al 2018  Chau et al, 2019 |  |  |  |  |  |
| Jaya et al., (2017) | 2017 | Cross-sectional | Adult community sample | Loneliness | Depressive symptoms & positive psychotic symptoms | Chau et al 2019  Erzen & Çikrikci, 2018 |  |  |  |  |  |
| **Psychosis – loneliness** | | | | | | |  |  |  |  |  |
| Badcock et al., (2015) | 2015 | Cross-sectional | Adults with psychosis | loneliness | Psychosis | Michalska da Rocha, et al 2018  Chau et al 2019 |  |  |  |  |  |
| Chrosek et al., (2016) | 2016 | Cross-sectional | Adults with psychotic disorder | Loneliness | Inpatient admissions | Lim et al 2018  Chau et al 2019 |  |  |  |  |  |
| Switaj et al., (2014) | 2014 | Cross-sectional | Adults with psychotic disorder | Loneliness | Depressive symptoms | Chau et al., 2019  Lim et al., 2018  Michalska da Rocha., et al 2018 |  |  |  |  |  |
| **Psychosis – loneliness & social support** | | | | | | |  |  |  |  |  |
| Roe et al., (2011) | 2011 | Cross-sectional | Adults with a diagnosis of a psychotic disorder | Social support & loneliness | Clinical recovery (symptoms severity & level of functioning) | Lim et al 2018  Michalska da Rocha, et al 2018  Chau et al 2019 |  |  |  |  |  |
| Sundermann et al., (2014) | 2014 | Cross-sectional | Patients with first-episode psychosis | Social support & loneliness | Psychotic symptoms | Michalska da Rocha, et al 2018  Chau et al 2019  Lim et al 2018 |  |  |  |  |  |
| **Psychosis – social support / social network** | | | | | | |  |  |  |  |  |
| Angell et al., (2002) | 2002 | Longitudinal | Young adults (18-25 years of age) with a diagnosis of schizophrenia, schizoaffective disorder or schizotypal personality disorder | Social network | Positive psychosis symptoms | Michalska da Rocha, et al 2018  Chau et al 2019 |  |  |  |  |  |
| Lipton et al., (1981) | 1981 | Cross-sectional | Adults with psychotic disorder | Social network & social support | Compared to comparison group | Gayer-Anderson & Morgan, 2013  Palumbo et al, 2015 |  |  |  |  |  |
| Thorup et al., (2006) | 2006 | Cross-sectional correlational and longitudinal | Adults with first episode psychosis (18-45 years of age) | Social Network | Psychotic symptoms and global functioning | Degnan et al, 2018  Gayer-Anderson and Morgan, 2013 |  |  |  |  |  |
| **PTSD – social support** | | | | | | |  |  |  |  |  |
| Abbas (2018) | 2018 | Cross-sectional | Participants with PTSD symptoms | Social support | Severity of PTSD symptoms | Blais et al, 2021  Zalta et al, 2020 |  |  |  |  |  |
| Balderrama-Durbin et al., (2013) | 2013 | Cross-sectional | Participants with PTSD symptoms | Social support | Severity of PTSD symptoms | Blais et al, 2021  Zalta et al, 2020 |  |  |  |  |  |
| Boul (2015) | 2015 | Cross-sectional | Participants with PTSD symptoms | Social support | Severity of PTSD symptoms | Blais et al, 2021  Zalta et al, 2020 |  |  |  |  |  |
| Britt et al., (2013) | 2013 | Cross-sectional | Participants with PTSD symptoms | Social support | Severity of PTSD symptoms | Blais et al, 2021  Zalta et al, 2020 |  |  |  |  |  |
| Burke et al., (2013) | 2010 | Cross-sectional | Participants with PTSD symptoms | Social support | PTSD symptoms | Scott et al, 2020  Zalta et al, 2020 |  |  |  |  |  |
| Campbell & Riggs (2015) | 2015 | Cross-sectional | Participants with PTSD symptoms | Social support | Severity of PTSD symptoms | Blais et al, 2021  Zalta et al, 2020 |  |  |  |  |  |
| Davis et al., (2015) | 2015 | Cross-sectional | Participants with PTSD symptoms | Social support | Severity of PTSD symptoms | Blais et al, 2021  Zalta et al, 2020 |  |  |  |  |  |
| Dempsey (2001) | 2001 | Cross-sectional | Participants with PTSD symptoms | Social support | Severity of PTSD symptoms | Blais et al, 2021  Zalta et al, 2020 |  |  |  |  |  |
| Dryden (2012) | 2012 | Cross-sectional | Participants with PTSD symptoms | Social support | Severity of PTSD symptoms | Blais et al, 2021  Zalta et al, 2020 |  |  |  |  |  |
| Gradust et al., (2015) | 2015 | Cross-sectional | Participants with PTSD symptoms | Social support | Severity of PTSD symptoms | Blais et al, 2021  Zalta et al, 2020 |  |  |  |  |  |
| Herbert et al., (2018) | 2018 | Cross-sectional | Participants with PTSD symptoms | Social support | Severity of PTSD symptoms | Blais et al, 2021  Zalta et al, 2020 |  |  |  |  |  |
| Hoyt & Renshaw (2014) | 2014 | Cross-sectional | Participants with PTSD symptoms | Social support | Severity of PTSD symptoms | Blais et al, 2021  Zalta et al, 2020 |  |  |  |  |  |
| Hoyt et al., (2010) | 2010 | Cross-sectional | Participants with PTSD symptoms | Social support | Severity of PTSD symptoms | Blais et al, 2021  Zalta et al, 2020 |  |  |  |  |  |
| Jacques-Tiura., (2010) | 2010 | Cross-sectional | Participants with PTSD symptoms | Social support | Severity of PTSD symptoms | Zalta et al, 2020  Tirone et al, 2021 |  |  |  |  |  |
| Kehle et al., (2010) | 2010 | Cross-sectional | Participants with PTSD symptoms | Social support | Severity of PTSD symptoms | Blais et al, 2021  Zalta et al, 2020 |  |  |  |  |  |
| King et al., (2006) | 2006 | Cross-sectional | Participants with PTSD symptoms | Social support | Severity of PTSD symptoms | Blais et al, 2021  Zalta et al, 2020 |  |  |  |  |  |
| Kline et al., (2013) | 2013 | Cross-sectional | Participants with PTSD symptoms | Social support | Severity of PTSD symptoms | Blais et al, 2021  Zalta et al, 2020 |  |  |  |  |  |
| Koster (2009) | 2009 | Cross-sectional | Participants with PTSD symptoms | Social support | Severity of PTSD symptoms | Blais et al, 2021  Zalta et al, 2020 |  |  |  |  |  |
| Laws et al., (2016) | 2016 | Cross-sectional | Participants with PTSD symptoms | Social support | Severity of PTSD symptoms | Blais et al, 2021  Zalta et al, 2020 |  |  |  |  |  |
| Lisman et al., (2017) | 2017 | Cross-sectional | Participants with PTSD symptoms | Social support | Severity of PTSD symptoms | Blais et al, 2021  Zalta et al, 2020 |  |  |  |  |  |
| Lubens & Silver (2019) | 2019 | Cross-sectional | Participants with PTSD symptoms | Social support | Severity of PTSD symptoms | Blais et al, 2021  Zalta et al, 2020 |  |  |  |  |  |
| Luciano & McDevitt-Murphy (2017) | 2017 | Cross-sectional | Participants with PTSD symptoms | Social support | Severity of PTSD symptoms | Blais et al, 2021  Zalta et al, 2020 |  |  |  |  |  |
| Lueger-Schuster et al., (2014) | 2014 | Cross-sectional | Participants with PTSD symptoms | Social support | Severity of PTSD symptoms | Tirone et al, 2021  Zalta et al, 2020 |  |  |  |  |  |
| Mendoza (2015) | 2015 | Cross-sectional | Participants with PTSD symptoms | Social support | Severity of PTSD symptoms | Blais et al, 2021  Zalta et al, 2020 |  |  |  |  |  |
| Moore et al., (2017) | 2017 | Cross-sectional | Participants with PTSD symptoms | Social support | Severity of PTSD symptoms | Blais et al, 2021  Zalta et al, 2020 |  |  |  |  |  |
| Muller & Lemieux (2000) | 2000 | Cross-sectional | Participants with PTSD symptoms | Social support | Severity of PTSD symptoms | Tirone et al, 2021  Zalta et al, 2020 |  |  |  |  |  |
| Nayback-Beebe & Yoder (2011) | 2011 | Cross-sectional | Participants with PTSD symptoms | Social support | Severity of PTSD symptoms | Blais et al, 2021  Zalta et al, 2020 |  |  |  |  |  |
| Palo & Gilbert (2015) | 2015 | Cross-sectional | Participants with PTSD symptoms | Social support | Severity of PTSD symptoms | Tirone et al, 2021  Zalta et al, 2020 |  |  |  |  |  |
| Pietrzak et al., (2009) | 2009 | Cross-sectional | Participants with PTSD symptoms | Social support | Severity of PTSD symptoms | Blais et al, 2021  Zalta et al, 2020 |  |  |  |  |  |
| Port (2002) | 2002 | Cross-sectional | Participants with PTSD symptoms | Social support | Severity of PTSD symptoms | Blais et al, 2021  Zalta et al, 2020 |  |  |  |  |  |
| Rivet (2012) | 2012 | Cross-sectional | Participants with PTSD symptoms | Social support | Severity of PTSD symptoms | Blais et al, 2021  Zalta et al, 2020 |  |  |  |  |  |
| Shaine (2016) | 2016 | Cross-sectional | Participants with PTSD symptoms | Social support | Severity of PTSD symptoms | Blais et al, 2021  Zalta et al, 2020 |  |  |  |  |  |
| Sprang & McNeil (1998) | 1998 | Cross-sectional | Participants with PTSD symptoms | Social support | Severity of PTSD symptoms | Scott et al, 2020  Zalta et al, 2020 |  |  |  |  |  |
| Steine et al., (2019) | 2019 | Cross-sectional | Participants with PTSD symptoms | Social support | Severity of PTSD symptoms | Tirone et al, 2021  Zalta et al, 2020 |  |  |  |  |  |
| Tackett (2011) | 2011 | Cross-sectional | Participants with PTSD symptoms | Social support | Severity of PTSD symptoms | Blais et al, 2021  Zalta et al, 2020 |  |  |  |  |  |
| Taft et al., (1999) | 1999 | Cross-sectional | Participants with PTSD symptoms | Social support | Severity of PTSD symptoms | Blais et al, 2021  Zalta et al, 2020 |  |  |  |  |  |
| Ullman & Relyea (2016) | 2016 | Cross-sectional | Participants with PTSD symptoms | Social support | Severity of PTSD symptoms | Tirone et al, 2021  Zalta et al, 2020 |  |  |  |  |  |
| Vernberg et al., (1996) | 1996 | Cross-sectional | Participants with PTSD symptoms | Social support | Severity of PTSD symptoms | Trickey et al, 2012  Allen et al, 2021 |  |  |  |  |  |
| Vogt et al., (2005) | 2005 | Cross-sectional | Participants with PTSD symptoms | Social support | Severity of PTSD symptoms | Blais et al, 2021  Zalta et al, 2020 |  |  |  |  |  |
| Weber (2012) | 2012 | Cross-sectional | Participants with PTSD symptoms | Social support | Severity of PTSD symptoms | Blais et al, 2021  Zalta et al, 2020 |  |  |  |  |  |
| Whalen (2011) | 2011 | Cross-sectional | Participants with PTSD symptoms | Social support | Severity of PTSD symptoms | Blais et al, 2021  Zalta et al, 2020 |  |  |  |  |  |
| Wilcox (2012) | 2012 | Cross-sectional | Participants with PTSD symptoms | Social support | Severity of PTSD symptoms | Blais et al, 2021  Zalta et al, 2020 |  |  |  |  |  |
| Wilson & Scarpa (2014) | 2014 | Cross-sectional | Participants with PTSD symptoms | Social support | Severity of PTSD symptoms | Tirone et al, 2021  Zalta et al, 2020 |  |  |  |  |  |
| Wolfe et al., (1998) | 1998 | Cross-sectional | Participants with PTSD symptoms | Social support | Severity of PTSD symptoms | Blais et al, 2021  Zalta et al, 2020 |  |  |  |  |  |
| Woodward et al., (2018) | 2018 | Cross-sectional | Participants with PTSD symptoms | Social support | Severity of PTSD symptoms | Blais et al, 2021  Zalta et al, 2020 |  |  |  |  |  |
| Wooten (2012) | 2012 | Cross-sectional | Participants with PTSD symptoms | Social support | Severity of PTSD symptoms | Blais et al, 2021  Zalta et al, 2020 |  |  |  |  |  |
| **Schizophrenia - loneliness** | | | | | | |  |  |  |  |  |
| Gallagher et al., (1995) | 1995 | Cross-sectional | Adults with schizophrenia | Loneliness | Hallucinations | Lim et al 2018  Chau et al 2019 |  |  |  |  |  |
| **Schizophrenia – social support or social network** | | | | | | |  |  |  |  |  |
| Cohen et al., (1997) | 1997 | Cross-sectional | Adults with schizophrenia/ a diagnosis of psychotic disorder | Social network | Schizophrenia | Lim et al 2018  Degnan et al, 2018 |  |  |  |  |  |
| Hamilton et al., (1989) | 1989 | Cross-sectional correlational | Adults with schizophrenia | Social Networks | Schizophrenia symptom severity (positive and negative) | Degnan et al, 2018  Palumbo et al, 2015 |  |  |  |  |  |
| Horan et al., (2006) | 2006 | Longitudinal | Adults with recent onset schizophrenia | Social network | Clinical functioning | Degnan et al, 2018  Gayer-Anderson&Morgan, 2013  Palumbo et al, 2015 |  |  |  |  |  |
| Thorup et al., (2007) | 2007 | Cross-sectional | Adults with first episode schizophrenia | Social support and social network | Premorbid functioning, age at first onset of symptoms & positive and negative symptoms | Gayer-Anderson and Morgan, 2013  Palumbo et al, 2015 |  |  |  |  |  |
| Tolsdorf (1976) | 1976 | Cross-sectional | Males with schizophrenia | Social support & social network | Schizophrenic symptoms | Gayer-Anderson and Morgan, 2013  Palumbo et al, 2015 |  |  |  |  |  |
| **Schizophrenia – social alienation** | | | | | | |  |  |  |  |  |
| Lindner at al., (2014) | 2014 | Experimental | Schizophrenia inpatients | Insula activation | Social alienation; agreeableness | Chau et al 2019  Michalska da Rocha, et al 2018 |  |  |  |  |  |
| **Schizophrenia & psychosis – social networks** | | | | | | |  |  |  |  |  |
| Becker et al., (1998) | 1998 | Cross-sectional | Adults with schizophrenia or psychosis | Social network & social support | Frequency of hospitalisation | Degnan et al, 2018  Palumbo et al, 2015 |  |  |  |  |  |
| **Bipolar disorder – social support** | | | | | | |  |  |  |  |  |
| Beyer et al., (2003) | 2003 | Cross-sectional | Older adults (50-89 years of age) and younger adults (18-49 years of age) with bipolar disorder | Social support | Time of onset of bipolar disorder | Greenberg et al, 2014  Studart et al, 2015 |  |  |  |  |  |
| Cohen et al., (2004) | 2004 | Longitudinal | Adults with bipolar I disorder | Social support | Bipolar disorder I symptoms | Greenberg et al, 2014  Studart et al., 2015  Wang et al., 2018 |  |  |  |  |  |
| Kulhara et al., (1999) | 1999 | Cross-sectional | Adults with bipolar disorder | Social support | Response to lithium measured by frequency of relapse | Studart et al, 2015  Greenberg et al, 2014 |  |  |  |  |  |
| Johnson et al., (1999) | 1999 | Longitudinal | Adults with bipolar I disorder | Perceived social support | Time to recovery, severity of depressive symptoms, severity of manic symptoms | Wang et al 2018  Greenberg et al, 2014  Studart et al, 2015 |  |  |  |  |  |
| Johnson et al., (2003) | 2003 | Longitudinal | Adults with bipolar disorder | Social support | Frequency of relapse | Greenberg et al, 2014  Studart et al, 2015 |  |  |  |  |  |
| O’Connell et al., (1985) | 1985 | Longitudinal | Adults with bipolar disorder | Social support | Response to lithium measured by frequency of relapse | Greenberg et al, 2014  Studart et al, 2015 |  |  |  |  |  |
| Romans and McPherson (1992) | 1992 | Cross-sectional | Adults with bipolar disorder | Social support | Duration of bipolar, prevalence of manic episodes | Greenberg et al, 2014  Studart et al, 2015 |  |  |  |  |  |
| Staner et al., (1997) | 1997 | Longitudinal | Adults with bipolar disorder | Social network | Relapse | Greenberg et al, 2014  Studart et al, 2015 |  |  |  |  |  |
| Strauss & Johnson (2006) | 2006 | Longitudinal | Adults with bipolar disorder | Social support | Manic symptoms | Greenberg et al, 2014  Studart et al, 2015 |  |  |  |  |  |
| Weinstock & Miller (2010) | 2010 | Longitudinal | Adults with bipolar disorder | Social support | Depressive symptoms | Greenberg et al, 2014  Studart et al, 2015 |  |  |  |  |  |
| **Mental ill-health in general (no specified mental health condition) – social capital or social support and/or social networks** | | | | | | |  |  |  |  |  |
| McIntyre et al., (2018) | 2018 | Cross-sectional | University students | Loneliness | Mental distress | Loades et al, 2020  Chau et al 2019 |  |  |  |  |  |
| **Other** | | | | | | |  |  |  |  |  |
| **Loneliness vs. social support: depression** | | | | | | |  |  |  |  |  |
| Hutcherson & Epkins (2009) | 2009 | Cross-sectional | Children/adolescents | Loneliness or social support | Depression symptoms | Rueger, 2016 (Social support & depression);  Loades et al, 2020 (Loneliness & depression) |  |  |  |  |  |
| Sjoberg et al., (2013) | 2013 | Longitudinal | All ages in general population (Gariepy et al, 2012)  Older adults (Courtin et al, 2017) | Loneliness or social support | Depression symptoms | Gariepy et al, 2012 (Social support & depression)  Courtin et al, 2017 (loneliness & depression) |  |  |  |  |  |
| **Social support: Depression vs. PTSD** | | | | | | |  |  |  |  |  |
| Banks & Weems (2014) | 2014 | Cross-sectional | Children/adolescents | Social support | Depression symptoms or PTSD | Rueger, 2016 (Social support & depression)  Allen et al, 2021 (Social support & PTSD) |  |  |  |  |  |
| Khamis (2008) | 2008 | Cross-sectional | Children/adolescents | Social support | Depression symptoms or PTSD | Rueger, 2016 (Social support & depression)  Allen et al, 2021 (Social support & PTSD) |  |  |  |  |  |
| Llabre & Hadi (1997) | 1997 | Cross-sectional | Children/adolescents | Social support | Depression symptoms or PTSD | Rueger, 2016 (Social support & depression)  Allen et al, 2021 (Social support & PTSD) |  |  |  |  |  |
| McQuaid (2005) | 2005 | Cross-sectional | Children/adolescents | Social support | Depression symptoms or PTSD | Rueger, 2016 (Social support & depression)  Allen et al, 2021 (Social support & PTSD) |  |  |  |  |  |
